# Supplementary material for: Pharmacological rescue of mutant p53 triggers spontaneous tumor regression via immune responses
Source: Cell Rep Med. 2025 Feb 21;6(3):101976. doi: 10.1016/j.xcrm.2025.101976 (PMC11970324; doi:10.1016/j.xcrm.2025.101976)
Supplement: Document S1. Figures S1–S5 [file mmc1.pdf]

Cell Reports Medicine, Volume *6*

Supplemental information

Pharmacological rescue of mutant p53 triggers spontaneous tumor regression via immune responses

Jiabing Li, Shuang Zhang, Baohui Wang, Yuting Dai, Jiale Wu, Dianjia Liu, Ying Liang, Shujun Xiao, Zhengyuan Wang, Jiaqi Wu, Derun Zheng, Xueqin Chen, Fangfang Shi, Kai Tan, Xianting Ding, Huaxin Song, Sujiang Zhang, and Min Lu

SUPPLEMENTARY INFORMATION

Figure S1

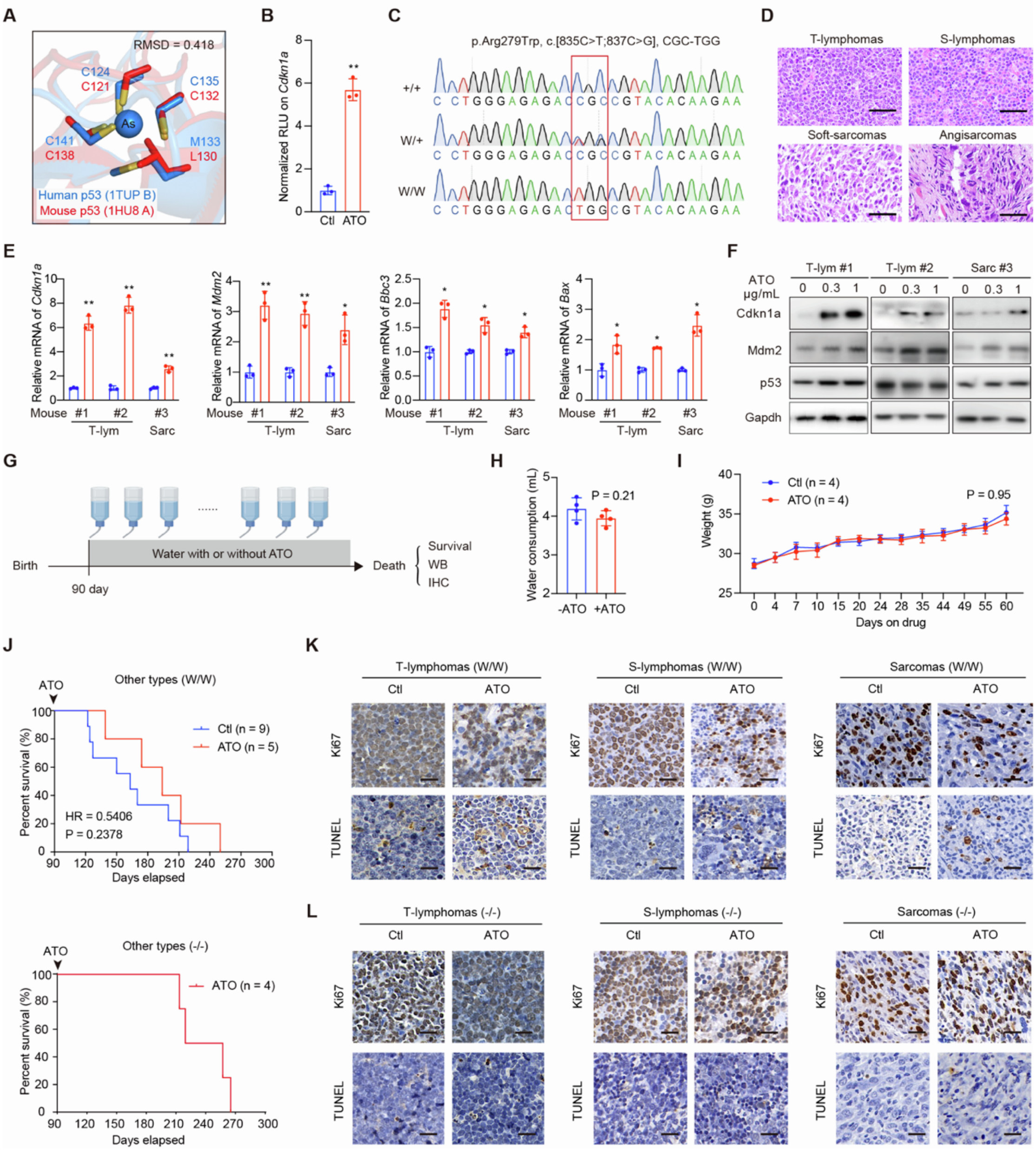

Figure S1. Pharmacologically rescued p53-R279W extended mice survival. Related to Figure 1.

(A) Close-up view of ABP-equivalent residues in the human wild-type p53 (1TUP chain B) and mouse wild-type p53 (1HU8 chain A) crystal structures. The ABP-equivalent residues are represented as sticks. The arsenic atom from the arsenic-bound G245S DBD (7DHY chain A) was superimposed onto human p53 and portrayed as a sphere.

The root-mean-square deviation (RMSD) calculation quantifies the differences between ABP residues in humans and mice.

(B) Luciferase reporter assay of mouse p53-R279W transcriptional activity on the mouse *Cdkn1a* promoter in H1299 cells upon treatment with 1 μg/ml ATO for 24h. Bar graphs show normalized relative light units (RLU).

(C) Sanger sequencing peak plots of the indicated genotypes in mice. Mutant codon positions were framed.

(D) H&E staining of the indicated tumors isolated from naturally dead W/W mice. Scale bar, 50 μm.

(E) mRNA levels of *Cdkn1a, Mdm2, Bbc3,* and *Bax* upon 1 μg/ml ATO treatment for 24h in the primary tumor cells derived from three naturally dead W/W mice.

(F) Immunoblotting of the indicated proteins in primary tumor cells derived from W/W mice upon treatment with 0, 0.3, and 1 μg/ml ATO for 48h.

(G) Schematic diagram of ATO treatment as in Figure 1D-1I.

(H) Water consumption by mice in the indicated groups (n = 4 mice per group).

(I) Body weight curves. The body weights of mice were measured every three days (n = 4 mice per group).

(J) Kaplan–Meier survival curves of W/W and −/− mice, with other tumor types, as shown in Figure 1D-1K.

(K-L) Representative immunohistochemical staining images of Ki67 and TUNEL in paraffin-embedded sections of T-lymphomas and S-lymphomas. Scale bars, 50 μm.

Bars represent mean ± SD, unpaired two-tailed Student’s t-test; In I, two-way ANOVA for multiple comparison testing. *$P < 0.05$, **$P < 0.01$.

Fig.S2

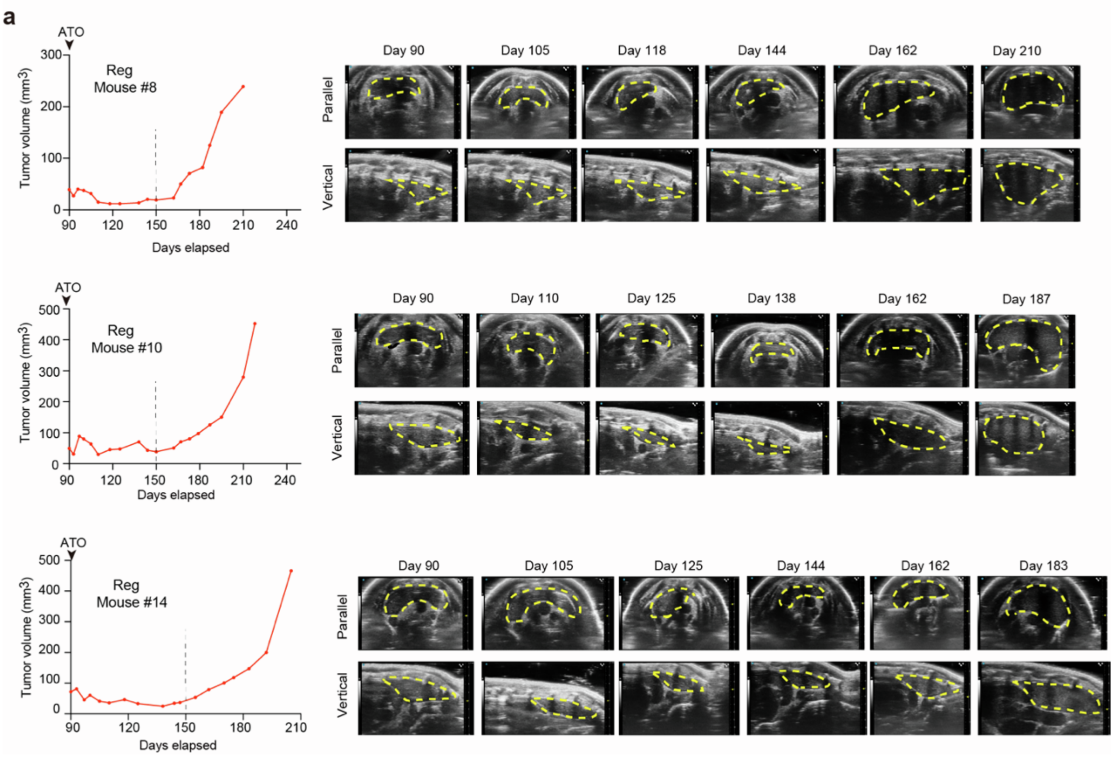

Figure S2. Rescued p53-R279W triggered regression of spontaneous lymphoma. Related to Figure 2.

(A) Examples of parallel and vertical ultrasound images of T-lymphomas in three representative mice from the regression group. The left panel of each image shows the calculated tumor volumes.

Fig.S3

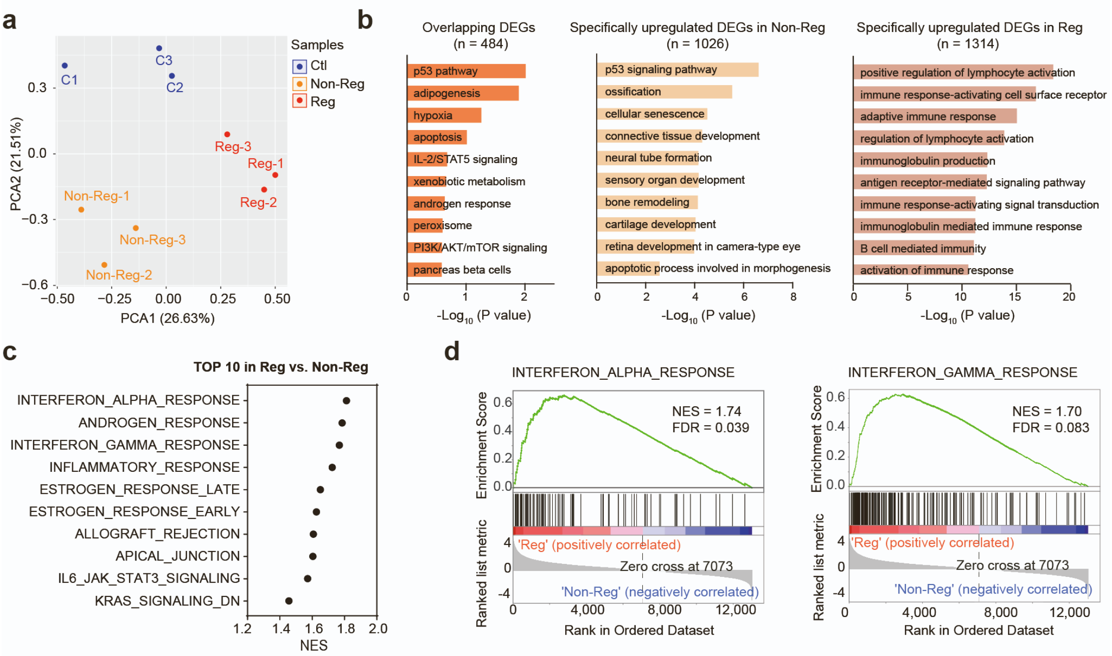

Figure S3. Lymphoma regression is associated with immune response. Related to Figure 3.

(A) 2D score plot of Principal Component Analysis (PCA) of tumors from three groups, as shown in Figure 3A.

(B) The full list of the top ten enriched pathways, as illustrated in Figure 3C.

(C) Dot plot showing the top ten pathways enriched in the Reg group identified by GSEA of hallmark gene sets compared to the Non-Reg group. NES: normalized enrichment score.

(D) GSEA plots of the enriched terms of “interferon alpha response” and “interferon gamma response” in C. NES and FDR (False discovery rate) are shown.

Fig.S4

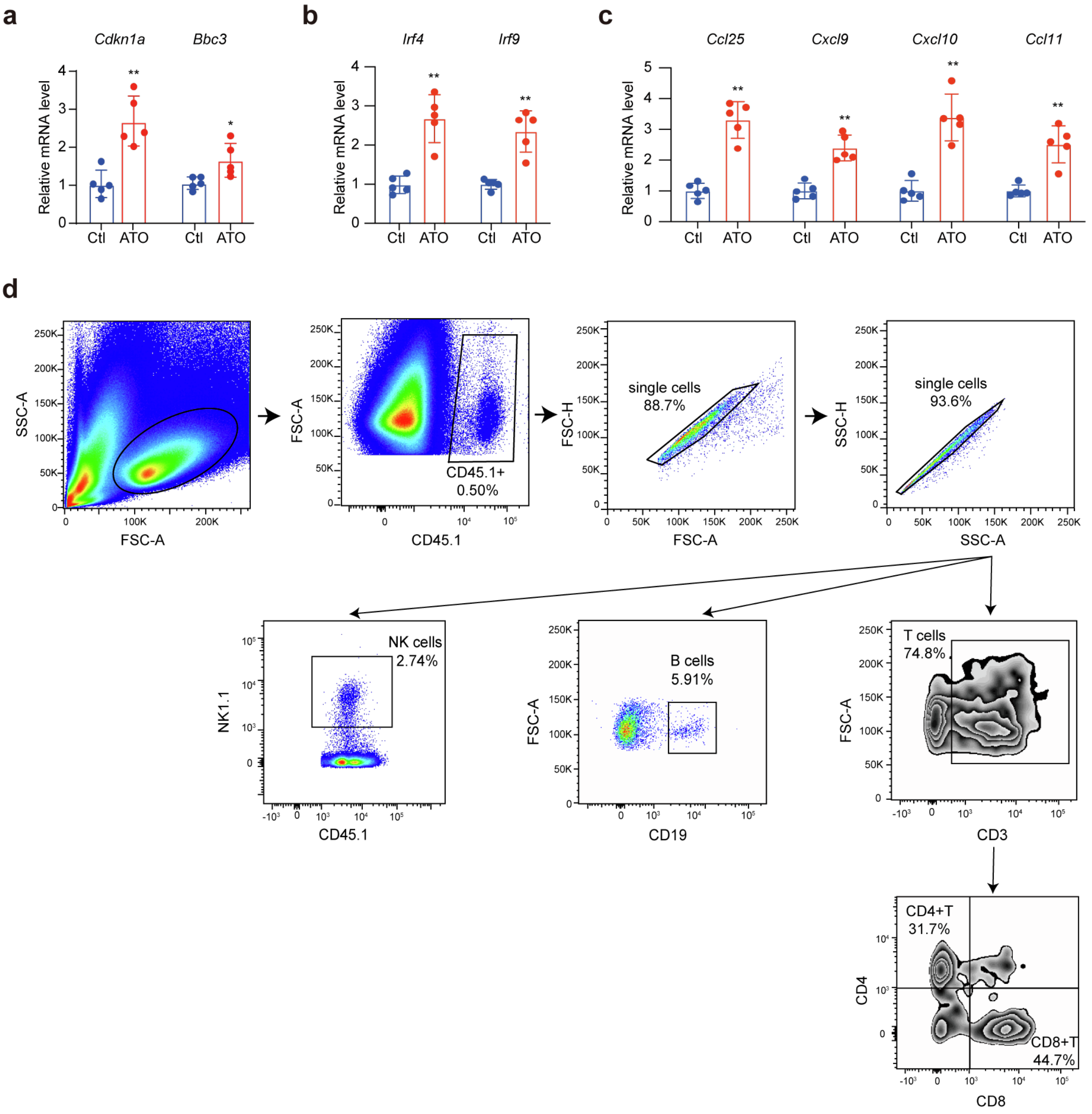

Figure S4. The anticancer immune response is largely dependent on the activation of CD8$^+$ T cells. Related to Figure 4.

(A-C) RT-qPCR validation of the relative mRNA levels of the indicated genes in the isolated tumors from Figure 4C. (A) p53 targets. (B) IFN pathway genes. (C) chemokine genes.

(D) Gating strategy of immune cells as in Figure 4E-4G.

Bars represent mean ± SD, unpaired two-tailed Student's t-test, *P < 0.05, **P < 0.01.

Fig.S5

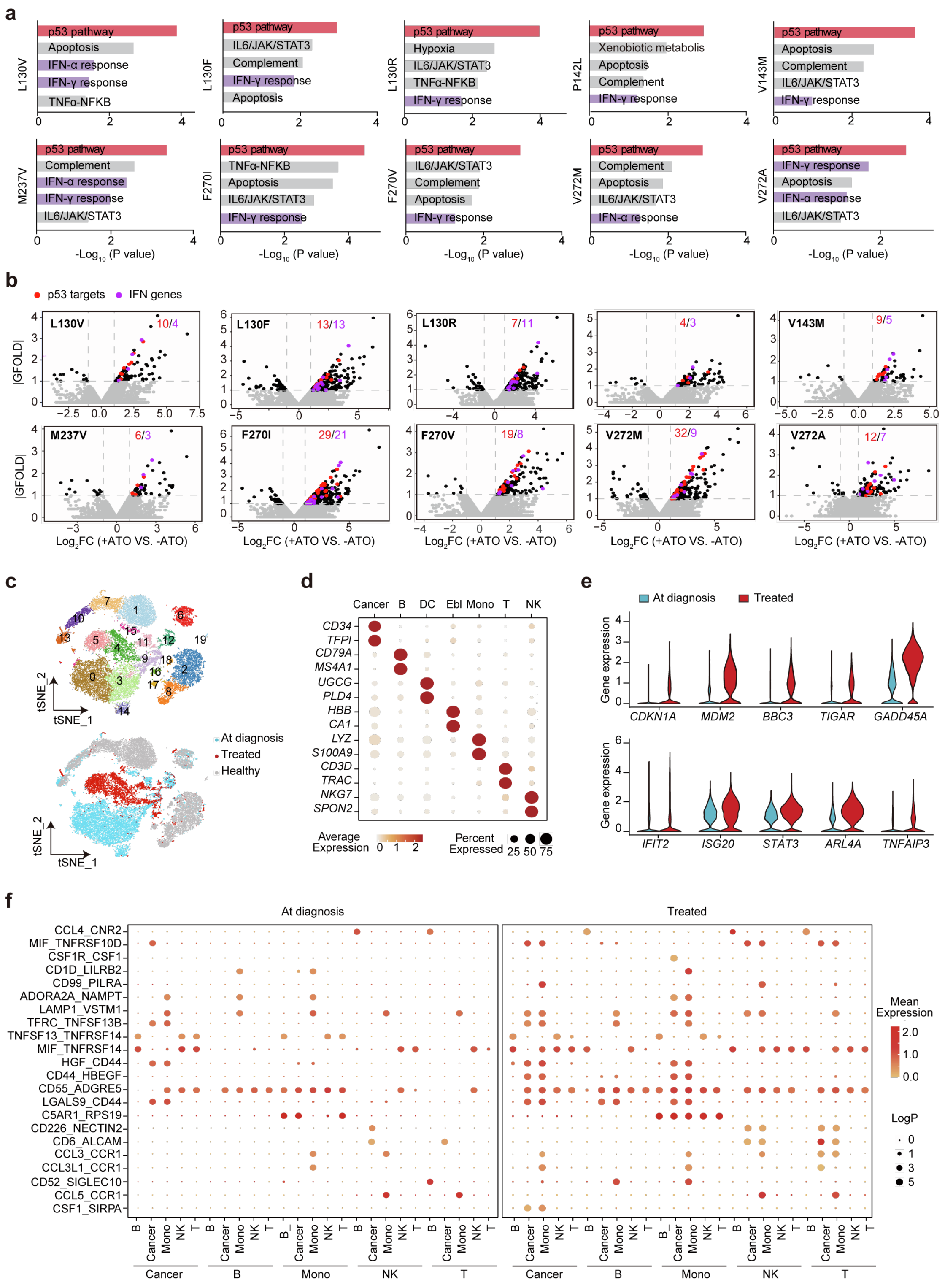

Figure S5. The broad applicability of rescued p53 mutants in triggering immune responses in human cells. Related to Figure 5.

(A) Top five pathways enriched in ATO-treated cells harboring the indicated type-1

p53 mutants. These enriched pathways were identified by the GSEA of hallmark gene sets for each mutant.

(B) Volcano plot showing DEGs (FC ≥ 2 or ≤ 0.5, GFOLD ≥ 1 or ≤ -1, FPKM ≥ 1) of the 10 indicated cell lines upon ATO treatment.

(C) Unsupervised *t*-SNE plot generated from datasets of PBMC samples in at-diagnosis, treated, and healthy humans.

(D) Dot plot of the marker genes for each classified cell type. These markers annotated 19 clusters into the six indicated cell types (cancer, cancer cells; B, B cells; T, T cells; NK, NK cells; Mono, monocytes; Ebl, erythroblasts; DC, dendritic cells).

(E) Violin plots showing the mRNA levels of representative p53 target genes (upper panel) and IFN pathway genes (lower panel) in cancer cells.

(F) Dot plot showing the expression of significant ligand-receptor pairs involved in the interaction among the six cell types before and after ATO treatment. P values are indicated by circle size (permutation test). The means of the average expression levels of interactions are indicated in color. The cell types below the line are ligand cells, and the cell types above the line are the corresponding receptor cells.
