# Supplementary material for: Pharmacological rescue of mutant p53 triggers spontaneous tumor regression via immune responses
Source: Cell Rep Med. 2025 Feb 21;6(3):101976. doi: 10.1016/j.xcrm.2025.101976 (PMC11970324; doi:10.1016/j.xcrm.2025.101976)
Supplement: Document S2. Article plus supplemental information [file mmc9.pdf]

Cell Reports Medicine

Article

Pharmacological rescue of mutant p53 triggers spontaneous tumor regression via immune responses

Graphical abstract

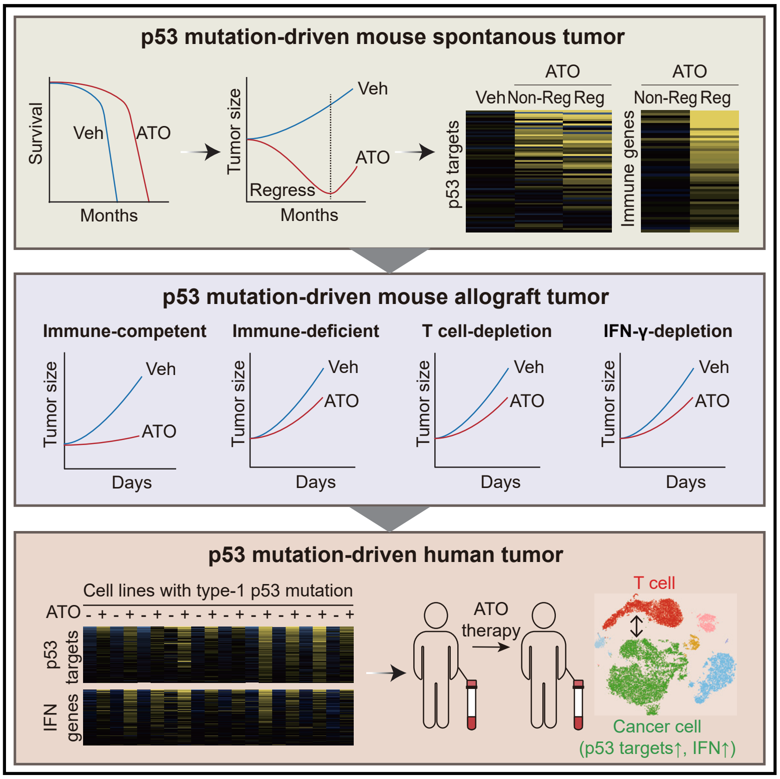

Highlights

- ATO triggers spontaneous tumor regression and extends survival of p53-mutant mice
- $CD8^+$ T cells and IFN-γ are crucial for ATO-induced tumor regression
- ATO triggers IFN signaling in p53-mutant cancer cell lines and primary cancer cells

Authors

Jiabing Li, Shuang Zhang, Baohui Wang, ..., Huaxin Song, Sujiang Zhang, Min Lu

Correspondence

dingxianting@sjtu.edu.cn (X.D.),
songhuaxin2017@163.com (H.S.),
zbruce.zhang@hotmail.com (S.Z.),
min.lu@shsmu.edu.cn (M.L.)

In brief

Li et al. explore the potential of pharmacological reactivation of mutant p53 in cancer treatment, showing that it effectively induces tumor regression and extends survival by restoring classic p53 tumor-suppressive functions and activating immune responses in mice, offering insights for tumor suppressor-based cancer immunotherapy.

Li et al., 2025, Cell Reports Medicine *6*, 101976
March 18, 2025 © 2025 The Author(s). Published by Elsevier Inc.
https://doi.org/10.1016/j.xcrm.2025.101976

CellPress

Cell Reports Medicine

CellPress
OPEN ACCESS

Article

Pharmacological rescue of mutant p53 triggers spontaneous tumor regression via immune responses

Jiabing Li,[1,4] Shuang Zhang,[2,4] Baohui Wang,[3,4] Yuting Dai,[1,4] Jiale Wu,[1,4] Dianjia Liu,[1] Ying Liang,[1] Shujun Xiao,[1] Zhengyuan Wang,[1] Jiaqi Wu,[1] Derun Zheng,[1] Xueqin Chen,[1] Fangfang Shi,[1] Kai Tan,[1] Xianting Ding,[2,*] Huaxin Song,[1,*] Sujiang Zhang,[1,*] and Min Lu[1,5,*]

[1]Shanghai Institute of Hematology, State Key Laboratory of Medical Genomics, National Research Center for Translational Medicine (Shanghai), Ruijin Hospital affiliated to Shanghai Jiao Tong University School of Medicine, Shanghai 200025, China

[2]State Key Laboratory of Systems Medicine for Cancer, Institute for Personalized Medicine and Med-X, Institute School of Biomedical Engineering Research, Shanghai Jiao Tong University, Shanghai, China

[3]The First Affiliated Hospital of Zhejiang Chinese Medical University, Zhejiang Chinese Medical University, Hangzhou 310006, China

[4]These authors contributed equally

[5]Lead contact

*Correspondence: dingxianting@sjtu.edu.cn (X.D.), songhuaxin2017@163.com (H.S.), zbruce.zhang@hotmail.com (S.Z.), min.lu@shsmu.edu.cn (M.L.)

https://doi.org/10.1016/j.xcrm.2025.101976

SUMMARY

Tumor suppressor p53 is the most frequently mutated protein in cancer, possessing untapped immune-modulating capabilities in anticancer treatment. Here, we investigate the efficacy and underlying mechanisms of pharmacological reactivation of mutant p53 in treating spontaneous tumors in mice. In the p53 R279W (equivalent to the human hotspot R282W) mouse model developing spontaneous tumors, arsenic trioxide (ATO) treatment through drinking water significantly prolongs the survival of mice, dependent on p53-R279W reactivation. Transient regressions of spontaneous T-lymphomas are observed in 70% of the ATO-treated mice, accompanied by interferon (IFN) response. In allograft models, the tumor-suppressive effect of reactivated p53-R279W is detectably reduced in both immunodeficient Rag1$^{-/-}$ and CD8^{+} T cell-depleted mice. ATO also activates the IFN pathway in human cancer cells harboring various p53 mutations, as well as in primary samples derived from the p53-mutant patient treated with ATO. Together, p53 could serve as an alternative therapeutic target for the development of immunotherapies.

INTRODUCTION

Tumor suppressor genes (TSGs) account for approximately half of the 568 reported mutational cancer driver genes.[1,2] Oncogenes account for about one-quarter.[2] This indicates that TSG inactivation is a common driver of cancer development. Although the cell-autonomous anticancer activities of TSGs are well-established, recent *in vivo* CRISPR screens in mouse models have surprisingly revealed the major and indispensable role of TSGs in adaptive immune system-triggered cancer cell elimination.[3] This highlights the key cell non-cell-autonomous immune-regulating activities of TSGs. However, *in vitro*, CRISPR screens in tissue cultures have only identified a limited number of TSGs that are indispensable for killing tumor cells by cytotoxic T cells.[4] This suggests that an integrated tumor immune microenvironment (TIME) is necessary for TSGs to fully execute their immune-regulating effects. Despite this growing knowledge, the immune-modulating activities of TSGs have not yet been applied to cancer treatment in clinics.

p53 is a key tumor suppressor in human cancers. Genetically modified mice with defective wild-type p53 function spontaneously develop multiple tumor types at an early stage.[5] Despite the extended time required for spontaneous tumor development in comparison to other models, these models play a crucial role in predicting treatment outcomes as they represent natural processes of tumor development and progression. Encouragingly, reintroducing wild-type p53 into p53-null or downregulated mice can effectively slow progression and even trigger spontaneous tumor regression.[6–11] This appears to be attributed to the coordination of the p53's conventional (cell-cycle arrest, senescence, and apoptosis) and unconventional (metabolic regulation, antioxidant function, genomic stabilization, and others) function,[12–14] as well as the non-cell-autonomous immune-modulating functions of p53.[10,11,15] Unfortunately, restoration of p53 function through gene therapy is currently challenging in clinical practice. Inactivated p53 mutants are present in around half of cancer cases, making pharmacological reactivation of mutant p53 using small molecules an attractive alternative.[16,17] To date, reactivation of mutant p53 in spontaneous tumor models remains an unexplored therapeutic avenue owing to the lack of a potent reactivating compound. Recently, we identified arsenic trioxide (ATO) as a potent mutant p53 reactivating compound.[18–20] ATO releases arsenic atoms and covalently binds to the arsenic-binding pocket (ABP), robustly

This is an open access article under the CC BY license (http://creativecommons.org/licenses/by/4.0/).

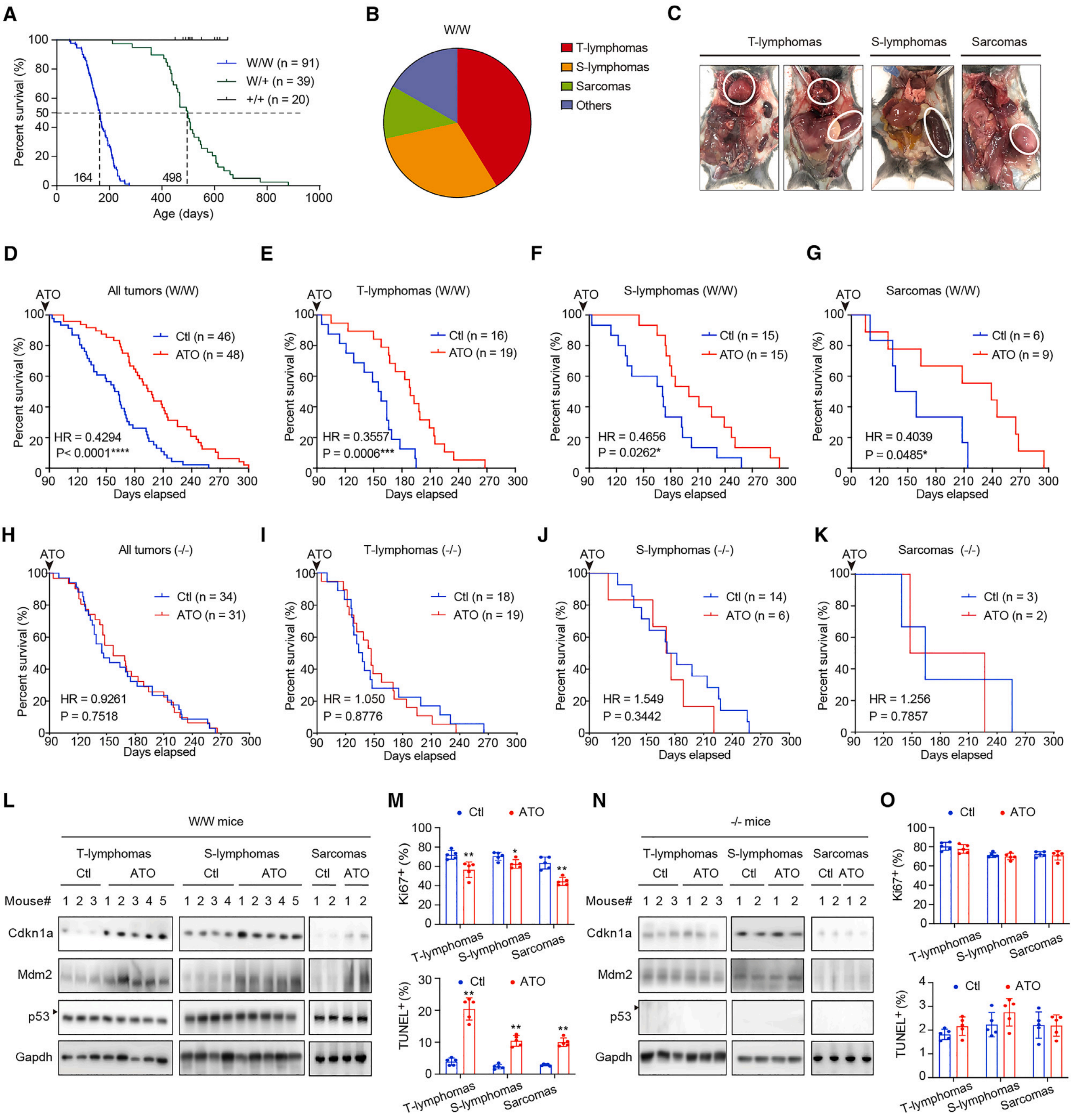

Figure 1. Pharmacologically rescued p53-R279W extended mice survival

(A) Kaplan-Meier survival curves for the wild-type (+/+), heterozygous $p53^{R279W/+}$ (W/+), and homozygous $p53^{R279W/R279W}$ (W/W) mice.

(B) Tumor spectrum of W/W mice.

(C) Macroscopic and phenotypic characteristics of representative spontaneous tumors in W/W mice.

(D–G) Kaplan-Meier survival curves of W/W mice treated with or without ATO (35 mg ATO in 1 L drinking water) from day 90 until natural death were analyzed. (D) Mice with all tumor types. (E) Mice with T-lymphomas. (F) Mice with S-lymphomas. (G) Mice with sarcomas.

(H–K) Kaplan-Meier survival curves of p53-null ($p53^{-/-}$, –/–) mice treated with or without ATO, as in (D)–(G) were compared.

(L) Immunoblotting of Cdkn1a, Mdm2, and p53 in T-lymphomas, S-lymphomas, and sarcomas isolated from natural-death W/W mice in (E) and (F). The molecular weight marked by the arrow is 55 kDa.

(M) Percentages of $Ki67^+$ and $TUNEL^+$ cells in immunohistochemistry-stained T-lymphomas, S-lymphomas, and sarcomas isolated from natural-death W/W mice in (E) and (F) (*n* = 5 mice per group).

(legend continued on next page)

strengthening the interaction between loop-sheet-helix (LSH) and β sandwich motifs, thereby stabilizing the p53 structure.[18] By doing so, ATO effectively rescues 390 structural p53 mutants, with a high rescue potency for the temperature-sensitive (TS) subtype of structural mutants.[19–21]

In this study, we demonstrated that pharmacological rescue of the TS-type p53 hotspot mutation R282W using ATO can lead to spontaneous tumor regression and significantly prolong the overall survival of mice, accompanying the activation of the immune response. Therefore, we propose TSGs as alternative targets for anticancer immune therapies.

RESULTS

Pharmacologically rescued p53-R279W extended mice survival

Among the six classic human p53 hotspot mutations, ATO most potently rescued the TS structural mutation R282W. However, the mouse model with knockin R279W (corresponding to human R282W) has not been previously reported.[18] ATO releases arsenic atoms and binds to ABP, which consists of Cys124-Met133-Cys135-Cys141 in human p53, with Met133 being substituted by Leu133 in mouse p53. From a structural viewpoint, the mouse Leu substitution did not alter the geometry of the ABP pocket or the orientations of the four arsenic-binding residues, thereby providing a possibility of rescuing mouse p53-R279W by ATO (Figure S1A). The transactivation activity of mouse p53-R279W was significantly rescued by ATO on the mouse *Cdkn1a* promoter, a representative p53 target (Figure S1B). With these validations, the R279W mutation was knocked into the *p53* gene locus of C57BL/6 mice to evaluate the treatment efficacy of pharmacological rescue of mutant p53 (Figure S1C).

As expected, homozygous $p53^{R279W/R279W}$ (W/W) and heterozygous $p53^{R279W/+}$ (W/+) mice displayed a significant decrease in the median survival time compared to wild-type mice, with respective median survival times of 164, 498, and >600 days (Figure 1A). Due to the diversity of individual mice and the complexity of spontaneous tumors, the W/W mice had relatively different overall survival ranging from 49 to 275 days (Figure 1A). The most common tumors spontaneously occurring in W/W mice were lymphomas, including thymic-lymphomas (T-lymphomas), which included those that were restricted macroscopically to the thymus, as well as those that spread to the liver and spleen, and spleen-lymphomas (S-lymphomas) characterized by massive hepatomegaly and splenomegaly without thymic involvement[22] (Figures 1B and S1D showing H&E staining). Sarcomas (including osteosarcomas, angiosarcoma, and fibrosarcoma), and other tumor types such as adenocarcinoma, were also observed.

Next, we investigated the potential of the pharmacological rescue of p53-R279W by ATO to improve the survival of mice. We first validated the effectiveness of ATO in rescuing R279W in cultured primary cell lines generated from spontaneous tumors of W/W mice. Treatment with 1 μg/mL ATO significantly upregulated the representative p53 targets *Cdkn1a*, *Mdm2*, *Bbc3*, and *Bax* at the mRNA levels (Figure S1E) and Cdkn1a and Mdm2 at the protein levels (Figure S1F). Based on these *in vitro* experiments, ATO was administered to W/W mice in their drinking water at a final concentration of 35 mg/L from day 90 until natural death (Figure S1G). ATO treatment did not significantly affect the water intake or body weight of mice (Figures S1H and S1I) but significantly prolonged the survival of mice with spontaneous tumors, with a hazard ratio (HR) of 0.4294 (Figure 1D, $p < 0.0001$). The ATO-treated mice had overall survival ranging from 105 to 300 days (Figure 1D). T-lymphoma-, S-lymphoma-, and sarcoma-bearing mice all experienced significant survival benefits with ATO treatment (Figures 1E–1G). Although mice with other rare tumor types exhibited extended survival after ATO treatment, the effects were not statistically significant (Figure S1J), presumably due to the limited number of mice. Notably, p53-null ($p53^{-/-}$) mice, with any type of spontaneous tumor, did not show significantly extended survival under the same ATO treatment, supporting the dependence of p53-R279W on the therapeutic efficacy of ATO (Figures 1H–1K and S1J lower panel).

To confirm that the efficacy of ATO is associated with the rescue of p53-R279W, tumors were isolated from ATO-treated and untreated W/W mice; immunoblotting showed that T-lymphomas, S-lymphomas, and sarcomas expressed upregulated Cdkn1a and Mdm2 in ATO-treated mice (Figure 1L). Immunohistochemical staining further revealed that ATO treatment significantly decreased the percentage of proliferating (Ki67-positive) cells and increased the percentage of apoptotic (TUNEL-positive) cells (Figures 1M and S1K). In T-lymphomas, S-lymphomas, and sarcomas isolated from ATO-treated $p53^{-/-}$ mice, Cdkn1a or Mdm2 was not upregulated (Figure 1N), without signs of cell proliferation inhibition or cell apoptosis induction (Figures 1O and S1L).

Rescued p53-R279W triggered regression of spontaneous lymphoma

Owing to the promising efficacy of ATO in treating W/W mice, we monitored the changes in spontaneous T-lymphomas (the most prevalent tumor type) throughout the tumor-bearing lifespan using high-resolution ultrasound imaging (Figure 2A). Mice harboring 50–100 mm^3 spontaneous tumors on day 90 after birth were selected, following ATO treatment in their drinking water. To better differentiate the 10 ATO-treated mice, we grouped them into the non-regression group (“Non-Reg” group) and regression group (“Reg” group) according to the appearance of tumor regression on day 150. Notably, grouping results based on any day during days 103–157 were similar, and thus we selected day 150 as the time point of defining tumor regression (Figure 2B; tumor examples are shown in Figures 2C and S2A). As a result, the 10 ATO-treated mice were divided into the“Non-Reg” (n = 5) and “Reg” (n = 5) groups. In the “Non-Reg” group, 2 mice ever experienced tumor regression during days 90–150. Among the total 7 mice that ever experienced

(N and O) Immunoblotting of the indicated protein and percentages of the indicated cells in immunohistochemistry staining for T-lymphomas, S-lymphomas, and sarcomas isolated from natural-death −/− mice in (L) and (M) (n = 5 mice per group).
Bars represent mean ± SD, unpaired two-tailed Student’s t test, *$p < 0.05$. **$p < 0.01$.

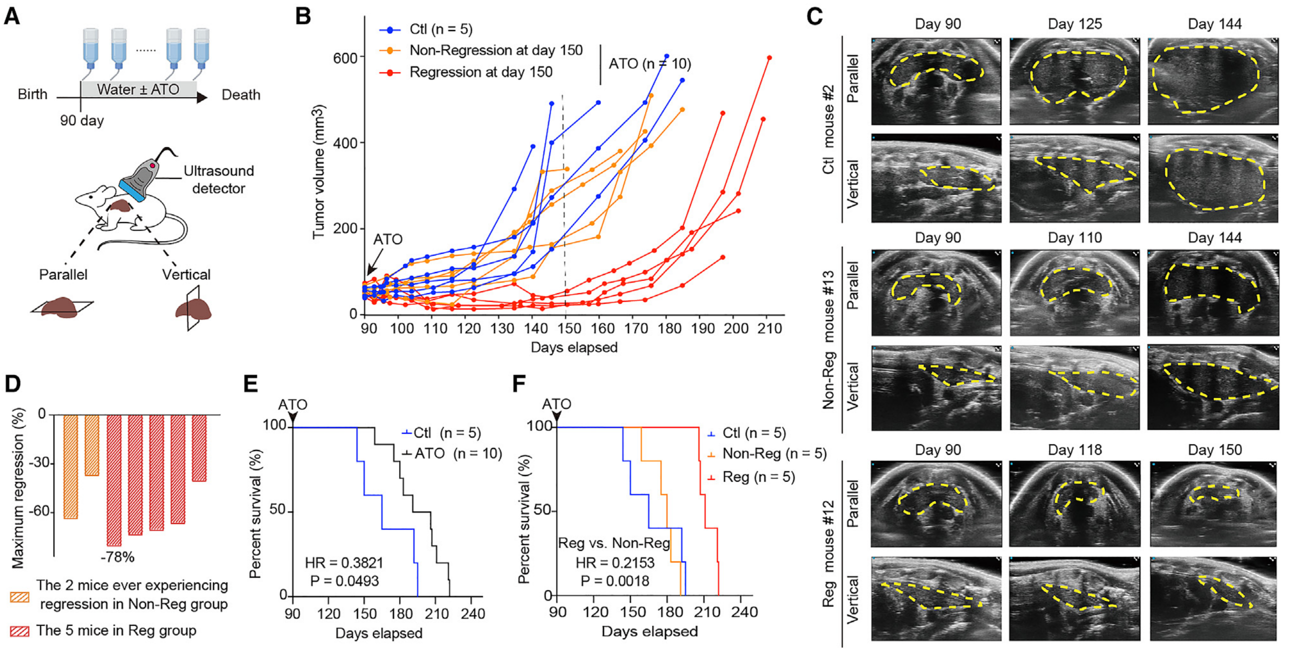

Figure 2. Rescued p53-R279W triggered regression of spontaneous lymphoma

(A–C) W/W T-lymphomas tumor monitoring using high-resolution ultrasound imaging. At day 90 after birth, W/W mice with T-lymphomas ranging from 50 to 100 mm^3 were selected and divided into groups receiving water with or without ATO until natural death. The tumors were scanned both parallel and vertically at specified time points. (A) Schematic diagram of the ultrasound imaging experiment. (B) Time-dependent growth curves of T-lymphomas. ATO-treated mice were categorized as regression (Reg, red lines) and non-regression (Non-Reg, yellow lines) based on their tumor regression status on day 150 (control [Ctl]: $n = 5$ mice; ATO-treated: $n = 10$ mice). (C) Representative ultrasound images of parallel and vertical T-lymphomas in the indicated group.

(D) Maximum regression rate of tumor volume in the seven mice that experienced regression in (B).

(E) Kaplan-Meier survival curves of mice treated with or without ATO.

(F) Kaplan-Meier survival curves of the indicated mice ($n = 5$ mice per group).

$^*p < 0.05$, $^{**}p < 0.01$.

regression (2 from the "Non-Reg" group and 5 from the "Reg" group), the maximum tumor volume regression rate was 78% (Figure 2D). The different responses among the treated 10 mice are possibly caused by the diversity of the individual mice, the complexity of the spontaneous tumors, and relatively long experiment durations in living animals, as in the survival experiments (Figures 1C and 1D). The 10 ATO-treated mice had significantly longer survival than the 5 ATO-untreated mice (Figure 2E, HR = 0.3821, $p = 0.0493$). In the ATO-treated group, the 5 mice in the regression group further had significantly longer survival compared to the mice in the non-regression group (Figure 2F, HR = 0.2153, $p = 0.0018$). All the mice in the experiment had died by day 222.

Lymphoma regression is associated with immune response

To investigate the mechanism underlying the observed tumor regression, W/W mice with T-lymphoma were administered as in the ultrasound imaging experiment. Tumor tissues were isolated from mice upon 60-day ATO treatment (day 150 after birth; three mice from each of the non-regression and regression groups) and subjected to RNA sequencing (RNA-seq) (Table S1). In the principal-component analysis (PCA) that assessed the clustering nature of the sequenced samples, samples from each group clustered together and showed a good correlation (Figure S3A). As expected, the established p53 target genes[23] were globally upregulated in the ATO-treated group, including the regression and non-regression groups, compared with the ATO-untreated group (Figure 3A). This was validated by determining the expression of the representative p53 targets *Trp53inp1*, *Btg2*, *Mdm2*, and *Cdkn1a* using reverse-transcription quantitative PCR (RT-qPCR) (Figure 3B). Thus, the conventional cell-autonomous functions of p53 should contribute to the tumor suppression function of ATO in both non-regression and regression mice; however, they are insufficient to explain the better treatment outcomes in regression mice. We next focused on the differentially expressed genes (DEGs; fold change [FC] ≥ 2 or ≤ 0.5, $p < 0.05$, fragments per kilobase million [FPKM] ≥ 1) in regression and non-regression groups compared to the ATO-untreated group. A total of 1,798 and 1,510 upregulated DEGs were found in the regression and non-regression groups, respectively, with 484 overlapping genes (Figure 3C). As expected, the overlapping 484 genes significantly enriched in the "p53 Pathway" (Figures 3C and S3B). The 1,026 DEGs specifically upregulated in the non-regression group were also significantly enriched in the "p53 signaling pathway." Intriguingly, 1,314 DEGs specifically upregulated in the regression group were highly enriched in a variety of immune response-related

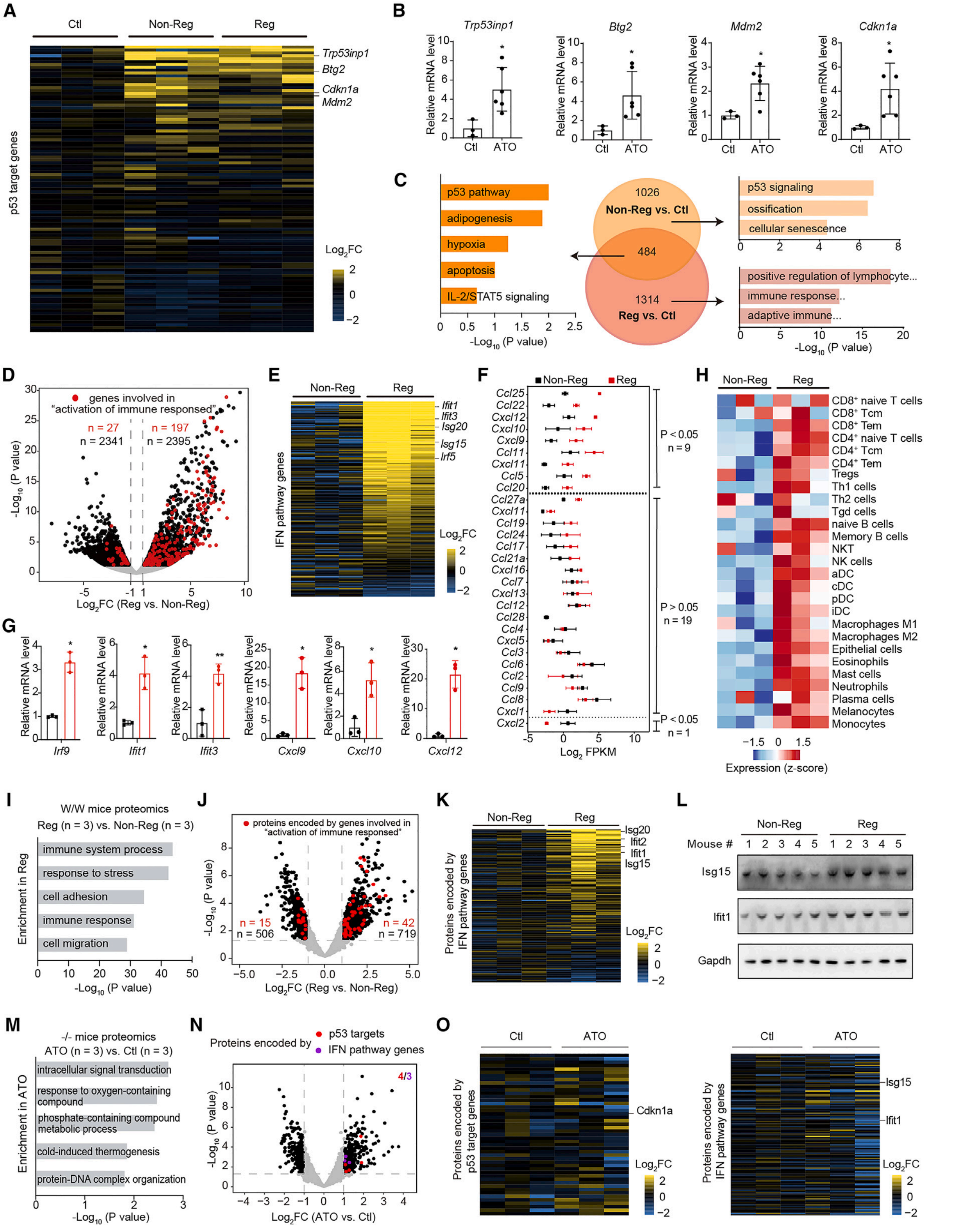

Figure 3. Lymphoma regression is associated with immune response

(A) Heatmap showing the relative expression levels of the established p53 targets in T-lymphomas isolated from W/W mice on day 150 after birth. The mice were treated as in the ultrasound imaging experiment, and the RNA-seq data were used for analysis (*n* = 3 mice per group).

(legend continued on next page)

pathways, including "positive regulation of lymphocyte activation" and "immune response-activating cell surface receptor." Thus, the immune response appears to be linked to p53 rescue-triggered tumor regression.

We next examined the immune response-related genes upregulated in the regression group. Compared to the non-regression group, 197 genes involved in the "activation of immune response" term in the Gene Ontology (GO) database were significantly upregulated in the regression group (Figure 3D). Gene set enrichment analysis (GSEA) highlighted the enrichment of genes involved in "interferon-alpha response (IFN-α response)," "interferon-gamma response (IFN-γ response)," and "inflammatory response" in the regression group using the molecular signatures database (MSigDB) hallmark gene set collection (Figures S3C and S3D showing two examples). Interferon signaling plays a crucial role in regulating antitumor immune response,[24] which may contribute to the observed ATO-triggered tumor regression. In the expression heatmap of the 244 genes involved in "IFN-α response" and "IFN-γ response" genes (herein termed IFN pathway genes), a large number of genes were upregulated in the regression group, including the key interferon-regulated genes *Irf5* and *Irf9*, interferon-inducible genes *Ifit1* and *Ifit3*, and interferon-stimulated genes *Isg15* (Figure 3E). It should be noted that *IRF5*,[25] *IRF9*,[26] and *ISG15*[27] are direct p53 targets. Chemokines are key cellular factors that mediate the entry of immune cells into tumors, thereby affecting tumor immunity and therapeutic outcomes.[28] Our RNA-seq analysis identified 29 detectable chemokines (FPKM > 0.1 in each sample), of which nine chemokines, including *Cxcl9*, *Cxcl10*, and *Cxcl11*, were significantly upregulated, whereas only *Cxcl2* was downregulated, in the regression group (Figure 3F). Importantly, these significantly upregulated chemokines have been previously reported to have antitumor immune-activating roles, with some being involved in the IFN pathway.[29–31] The upregulation of representative IFN pathway genes and chemokines in the regression group was confirmed using RT-qPCR (Figure 3G). Furthermore, we determined the composition of the immune landscape for the tumor isolated from both the regression and non-regression groups, by calculating the average expression levels of a set of signature genes associated with immune cells (Figure 3H). The analysis revealed that the regression group generally exhibited a higher level of immune cell infiltration compared to the non-regression group.

Proteins are direct executors of biological functions, and thus proteomics provides a more direct insight into biological processes compared to transcriptomics. To comprehensively characterize the responses triggered by ATO during tumor regression at the protein level, tumors from non-regression and regression groups ($n = 3$ in each group) were collected for liquid chromatography-tandem mass spectrometry (LC-MS/MS) proteomic determination (Table S2). Enrichment analysis, predicated on differential proteins (FC $\geq$ 2, $p < 0.05$), showed notable enrichment in immune response-related pathways within the regression group, particularly in the terms "immune system process" and "immune response" (Figure 3I). We next focused on the proteins involved in the "activation of immune response" term. 42 proteins associated with the term were significantly upregulated, whereas 15 were downregulated, in the regression group (Figure 3J). In addition, a number of proteins encoded by IFN pathway genes were observed to be upregulated in the regression group, including the representative Isg20, Ifit2, Ifit1, and Isg15 (Figure 3K). Immunoblotting validated these findings, showing detectably elevated expression levels of Isg15 and Ifit1 in the regression group tumors (Figure 3L). Collectively, the classic cell-autonomous activities of p53 (such as transactivating classic p53 targets) are evident in both non-regression and regression groups, while the non-cell-autonomous immune responses are preferentially observed in regression groups.

To clarify the dependence of immune activation in mutant p53 reactivation, T-lymphomas isolated from ATO-treated p53$^{-/-}$ mice were also collected for LC-MS/MS proteomics (Table S3). As a result, ATO administration in p53$^{-/-}$ mice did not activate the p53 pathway or immune-related pathways (Figure 3M). Among the 384 upregulated proteins after ATO administration, only 4 were p53 target proteins and 3 were IFN pathway-related proteins (Figure 3N). The heatmap further demonstrated that the

(B) RT-qPCR validation of the indicated p53 targets in T-lymphoma as in (A).
(C) Venn diagrams showing the distribution of unique upregulated differentially expressed genes (DEGs, FC $\geq$ 2 or $\leq$ 0.5, $p < 0.05$, FPKM $\geq$ 1 in RNA-seq) in tumors derived from non-regression and regression groups compared to the untreated group (Ctl). In total, 484 genes overlapped. Bar graphs show the top three pathways enriched in the functional enrichment analysis for the indicated DEGs (performed using g:Profiler).
(D–F) Comparison of gene expression in tumors isolated from Reg and Non-Reg groups in RNA-seq ($n = 3$ mice per group). (D) Volcano plot showing changes in gene expression between the Reg and Non-Reg groups. DEGs are shown in black, and DEGs involved in the "activation of immune response" term (GO: 0002253) are shown in red. Dashed lines indicate FC $\geq$ 2 or $\leq$0.5 and $p < 0.05$. (E) Heatmap showing the mRNA expression levels (Z score) of genes involved in the IFN pathway (genes involved in "IFN-α response" and "IFN-γ response" in MSigDB hallmark gene sets) in the tumors from the Non-Reg and Reg groups. (F) Scatterplot of Ccl and Cxcl chemokine gene expression in tumors from the Non-Reg and Reg groups.
(G) RT-qPCR validation of the indicated genes involved in the IFN pathway and chemokine genes in tumors from the Non-Reg and Reg groups.
(H) Heatmap showing abundance score of tumor-infiltrating immune cells in tumors from the Non-Reg and Reg groups.
(I–L) Proteomics analyses of T-lymphoma from Reg and Non-Reg groups ($n = 3$ mice per group). (I) Top five enriched pathways for differentially expressed proteins (FC $\geq$ 2, $p < 0.05$) in tumors (Reg vs. Non-Reg). (J) Volcano plot showing changes in protein expression between the Reg and Non-Reg groups. Altered proteins are shown in black, and altered proteins involved in the "activation of immune response" term are shown in red as shown in (D). Dashed lines indicate FC $\geq$ 2 or $\leq$0.5 and $p < 0.05$. (K) Heatmap showing the protein expression levels of genes involved in the IFN pathway in the indicated group as shown in (E). (L) Immunoblotting of the indicated proteins in tumors derived from the indicated mice.
(M–O) Proteomics analyses of T-lymphoma from $-/-$ mice treated with or without ATO ($n = 3$ mice per group). (M) Top five enriched pathways for differentially expressed proteins (FC $\geq$ 2, $p < 0.05$) of $-/-$ mice between the Ctl and ATO groups. (N) Volcano plot showing changes in protein expression of the indicated groups. Altered proteins are shown in black, and altered p53 targets and IFN pathway proteins are depicted in red and purple, respectively. (O) Heatmaps showing protein expression of established p53 targets (left panel) and IFN pathway (right panel) in the indicated groups.
Bars represent mean ± SD, unpaired two-tailed Student's t test. *$p < 0.05$, **$p < 0.01$.

CellPress
OPEN ACCESS

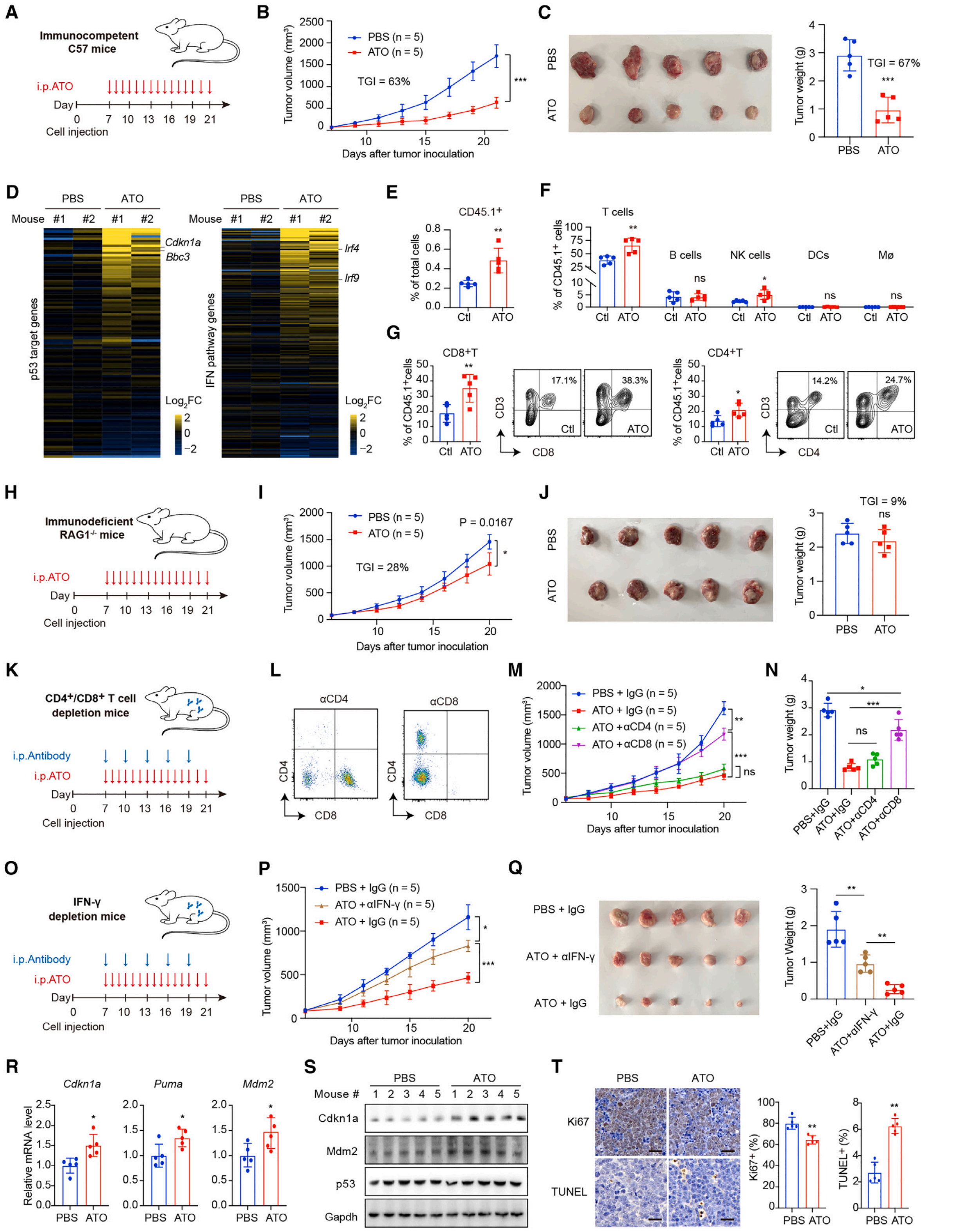

Figure 4. The anticancer immune response is partly dependent on the activation of CD8^{+} T cells

(A–G) ATO treatment in immunocompetent mice implanted with primary T-lymphoma cells derived from W/W mice. (A) Schematic diagram of ATO treatment in immunocompetent female C57BL/6 mice. The CD45.1 host mice were implanted with primary T-lymphoma cells derived from W/W mice. Mice were injected

(legend continued on next page)

proteins encoded by p53 target genes and IFN pathway genes are scarcely affected by ATO in p53$^{-/-}$ mice (Figure 3O). The aforementioned finding indicated that ATO-triggered immune response *in vivo* is dependent on the existence of the p53-R279W mutant.

The anticancer immune response is partly dependent on the activation of CD8^{+} T cells

To investigate the role of immune response in the antitumor effect of reactivated p53-R279W, we established three mouse models by transplanting primary T-lymphoma cells from W/W mice into immunocompetent and immunodeficient mice. Primary W/W T-lymphoma cells were subcutaneously injected into immunocompetent C57BL/6 mice on day 0, followed by daily intraperitoneal injection of 5 mg/kg ATO from day 7 (Figure 4A). ATO treatment significantly reduced tumor size and weight at the treatment endpoint, with tumor growth inhibition (TGI) values for tumor size and weight of 63% and 67%, respectively (Figures 4B and 4C). RNA-seq analysis demonstrated that ATO treatment globally upregulated a set of p53 targets (e.g., *Cdkn1a* and *Bbc3*) and IFN pathway genes (e.g., *Irf4* and *Irf9*) (Figure 4D; Table S4). We confirmed the upregulation of representative p53 targets, IFN pathway genes, and chemokines with antitumor immune-activating roles in ATO-treated tumors by RT-qPCR (Figures S4A–S4C). We also investigated immune cell infiltration in isolated tumors. A significant increase of CD45.1^{+} cells (representing the total tumor-infiltrating host immune cells) was observed in the tumors of the ATO-treated group (Figure 4E). CD45.1^{+} cells were differentiated into different subtypes of immune cells (Figure S4D), and it was observed that, among CD45.1^{+} cells, CD3^{+} T cells, and NK1.1^{+} natural killer (NK) cells, but not CD19^{+} B cells, CD11b^{+} CD11c^{+} dendritic cells (DCs), or CD11b^{+} F4/80^{+} macrophage cells, were significantly increased (Figure 4F). In the CD3^{+} T cells, both CD8^{+} T and CD4^{+} T cells were significantly increased (Figure 4G). This allograft model showed that the reactivation of p53-R279W was accompanied by the activation of immune responses and infiltration of immune cells.

To evaluate the contribution of the triggered immune response to the antitumor effect of reactivated p53-R279W, we injected primary W/W T-lymphoma cells into immunodeficient Rag1$^{-/-}$ mice, which lack functional B and T cells, followed by treatment as described earlier (Figure 4H). ATO treatment in this model resulted in lower extents of tumor size and weight reduction at the treatment endpoint compared to immunocompetent mice, with TGI values of 28% and 9%, respectively (Figures 4I and 4J). Therefore, B cells, T cells, or both are indispensable for the full tumor suppression function of reactivated p53-R279W.

Given that CD4^{+} T and CD8^{+} T cells, but not B cells, were highly infiltrated in ATO-treated tumors (Figures 4F and 4G), we explored the indispensability of CD4^{+} and CD8^{+} T cells, which are two key immune cell subsets that coordinate antitumor immune responses,[32] in the tumor suppression function of the reactivated p53-R279W. 200 μg αCD4, αCD8, or isotype-control immunoglobulin G (IgG) antibodies were intraperitoneally injected into immunocompetent C57BL/6 mice inoculated with W/W T-lymphoma cells every three days from the first ATO administration (Figure 4K). The efficacy of T cell depletion on day 21 was confirmed using fluorescence-activated cell sorting (FACS) (Figure 4L). The depletion of CD8^{+} T cells, but not CD4^{+} T cells, partly abrogated the tumor growth suppression function of ATO at the endpoint ($p < 0.001$ and $p > 0.05$, respectively) (Figure 4M). Consistently, the weight of the isolated tumors in the CD8^{+} T cell-depleting group, but not in the CD4^{+} T cell-depleting group, was significantly higher than that in the isotype-control IgG group ($p < 0.0001$ and $p > 0.05$, respectively) (Figure 4N). Above all, CD8^{+} T cells are partly indispensable for the tumor-suppressive function of the rescued p53-R279W in mice.

Given the enrichment of the IFN response signature in spontaneous and xenograft tumors after ATO treatment (Figures 3E and 4D), we next explored the role of the key IFN-γ in ATO's antitumor activities. IFN-γ was blocked through anti-IFN-γ antibody

intraperitoneally with ATO (5 mg/kg) or PBS daily from day 7 and were sacrificed on day 21. Tumor sizes were measured every three days ($n = 5$ mice per group).
(B) Time-dependent growth curves of tumor volume. (C) Photographs and weights of the isolated tumors. (D) Heatmap showing the relative gene expression of the established p53 targets and IFN pathway genes in the RNA-seq data of the tumors isolated from (B) ($n = 2$ mice per group). (E) The isolated tumors were cut into small pieces and mechanically disassociated into single-cell suspensions for FACS. Bar graphs show the percentage of host CD45.1^{+} cells infiltrating the tumors in the PBS and ATO groups. (F) Bar graphs show the percentages of tumor-infiltrating T cells, natural killer (NK) cells, B cells, dendritic cells (DCs), and macrophages (Mø) (relative to the total number of CD45.1^{+} cells) in the indicated groups. (G) Bar graphs show the percentages of tumor-infiltrating CD4^{+} T cells and CD8^{+} T cells (relative to the total number of CD45.1^{+} cells) in the indicated groups. Examples of CD4^{+} T and CD8^{+} T cell staining profiles are shown.
(H–J) ATO treatment in immunodeficient female Rag1$^{-/-}$ mice implanted with primary T-lymphoma cells derived from W/W mice. The experiments and calculations were performed as in (A)–(C) except that immunodeficient mice were used ($n = 5$ mice per group). (H) Schematic diagram of the experiment using immunodeficient Rag1$^{-/-}$ mice. (I) Time-dependent curves of tumor growth. (J) Tumor photographs and weights.
(K–N) ATO treatment in CD4^{+} and CD8^{+} T cell-depleted mice implanted with primary T-lymphoma cells derived from W/W mice. The experiments and calculations were performed as in (A)–(C), and T cells were depleted during ATO treatment ($n = 5$ mice per group). (K) Schematic diagram of the experiment using T cell depletion mice. For T cell depletion, αCD8 or αCD4 or isotype IgG (200 μg) was intraperitoneally injected at the indicated time points of each group. (L) CD4 and CD8 staining profiles of single-cell suspensions from the mouse spleen on day 21, as analyzed by FACS. (M and N) Time-dependent tumor growth curves and tumor weights.
(O–Q) ATO treatment mice upon IFN-γ blockade implanted with primary T-lymphoma cells derived from W/W mice. The experiments and calculations were performed as in (A)–(C), and the expression of IFN-γ was depleted during ATO treatment ($n = 5$ mice per group). (O) Schematic diagram of the experiment using IFN-γ depletion mice. IFN-γ or isotype IgG (200 μg) was intraperitoneally injected at the indicated time points of each group. (P) Time-dependent growth curves of tumor volume. (Q) Photographs and weights of the isolated tumors.
(R) mRNA levels of *Cdkn1a*, *Puma*, and *Mdm2* of tumors isolated from (I).
(S) Immunoblotting of Cdkn1a, Mdm2, and p53 of tumors isolated from (I).
(T) Representative immunohistochemical staining images and percentages of Ki67^{+} and TUNEL^{+} cells of tumors isolated from (I). Scale bars, 50 μm.
Bars represent mean ± SD, unpaired two-tailed Student's t test, *$p < 0.05$, **$p < 0.01$, ***$p < 0.001$.

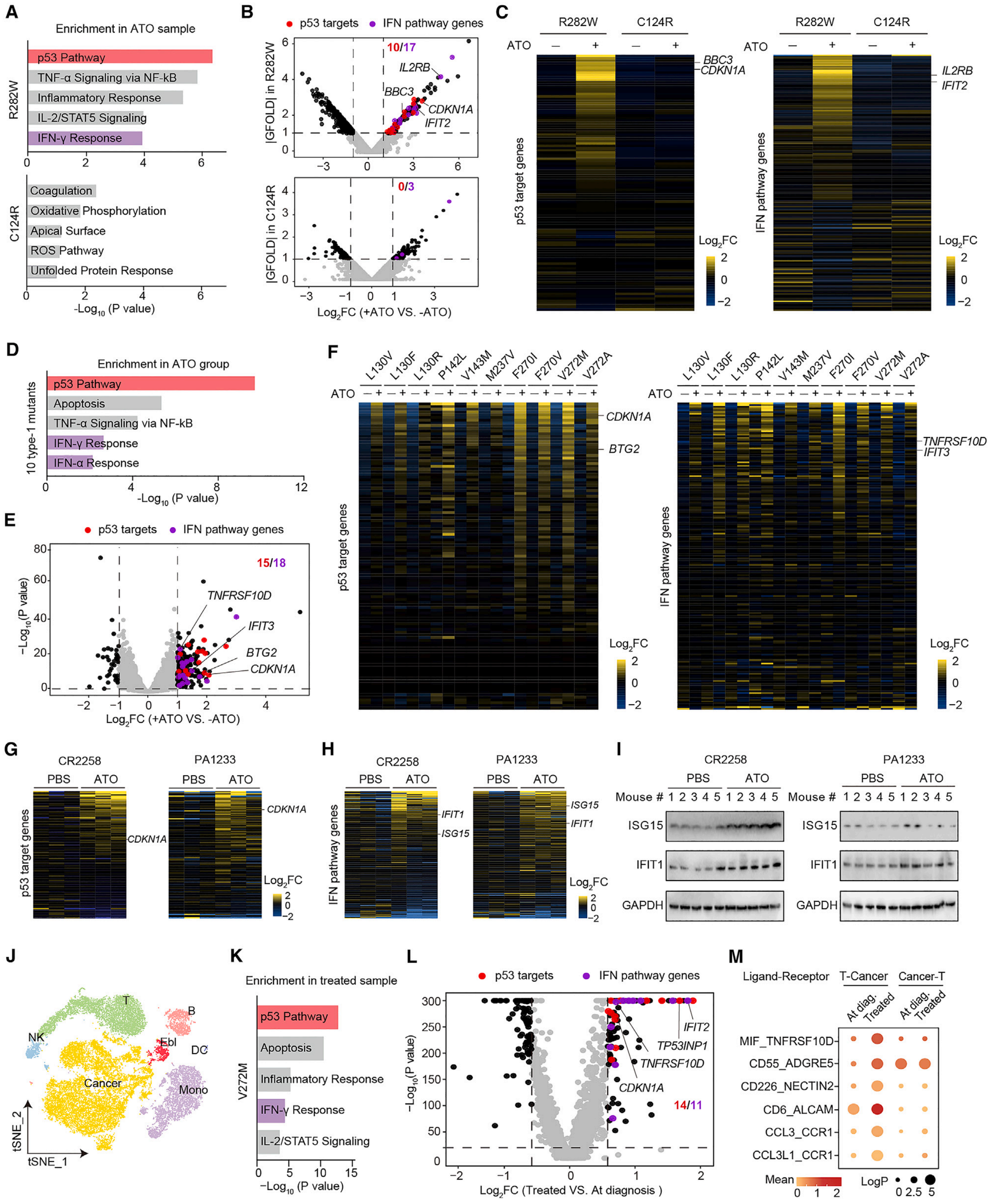

Figure 5. The broad applicability of rescued p53 mutants in triggering immune responses in human cells

(A–C) p53-null U937 cells infected with p53-R282W or p53-C124R and treated with 1 μg/mL ATO for 24 h, followed by RNA-seq analysis. (A) Top five enriched pathways identified by GSEA of hallmark gene sets in ATO-treated cells with the indicated p53 mutants. The p53 pathway is depicted as red bars, and the IFN

(legend continued on next page)

administration during ATO treatment in the immunocompetent C57 mice (Figure 4O). We observed that IFN-γ neutralization significantly impaired the antitumor effects of ATO, evidenced by a significant acceleration in tumor volume and tumor weight compared to the ATO + IgG group (Figures 4P and 4Q, $p < 0.005$ and $p < 0.01$, respectively), indicating that IFN-γ secretion is (partly) required to slow tumor growth upon ATO treatment.

Conventionally, p53 is reported to suppress tumors through cell-autonomous functions, representatively inducing cell apoptosis through transactivating pro-apoptotic targets and inducing cell-cycle arrest (also cell senescence) through transactivating targets such as *CDKN1A*. Indeed, we previously reported that ATO could effectively suppress tumor xenografts in various immunodeficient mouse models.[18,33] We thus determined whether these classic apoptosis and cell-cycle arrest functions were involved in the current ATO-treated immunodeficient Rag1$^{-/-}$ mice (Figure 4H). We assessed a series of classic p53 targets in tumors isolated from this mouse model. In the tumors isolated from mice in the ATO group, mRNA levels of *Cdkn1a* (related to cell-cycle arrest and senescence), *Puma* (related to apoptosis), and *Mdm2* (related to p53 negative self-regulation) significantly increased (Figure 4R). Protein analysis revealed elevated levels of Cdkn1a and Mdm2 in the ATO-treated tumors (Figure 4S). Immunohistochemistry detected significantly decreased cell proliferation (Ki67-positive) and increased apoptosis (TUNEL-positive) after ATO treatment (Figure 4T). These results explained the impaired but still significant tumor suppression ability of ATO in the immunodeficient Rag1$^{-/-}$ mice (Figure 4I), CD4$^+$/CD8$^+$ T cell depletion mice (Figure 4M), IFN-γ depletion mice (Figure 4P), and our previous studied mouse models.[18,33] Thus, the potent tumor suppression ability of ATO observed in the immunocompetent mouse model (Figure 4B) is likely caused by both the well-established classic cell-autonomous functions and currently observed non-cell-autonomous immune activation functions.

The broad applicability of rescued p53 mutants in triggering immune responses in human cells

We next investigated the potential of rescued p53 in triggering immune responses in human cancer cells. First, we generated isogenic U937 cell lines that expressed either human p53-R282W or p53-C124R, which cannot be rescued by ATO due to the mutation occurring on arsenic-bound Cys124.[21] These cell lines share identical genetic backgrounds, except for their p53 status, allowing any differences observed among them to be attributed to differences in p53 status. After ATO treatment and the followed RNA-seq analysis (Table S5), we found that ATO-treated transcriptome had the most profound changes in the "p53 pathway" and, in addition, "IFN-γ response" and other immune/inflammation-related pathways in cells harboring p53-R282W, as shown by GSEA analysis (Figure 5A). In contrast, no such pathway was enriched in p53-C124R cells. ATO treatment significantly upregulated 10 p53 targets and 17 IFN pathway genes in R282W cells, but only 0 and 3 genes in C124R cells of DEGs (FC ≥ 2 or ≤ 0.5, GFOLD ≥ 1 or ≤ -1, FPKM ≥ 1), respectively (Figure 5B). Heatmaps further revealed that a set of p53 targets including representative *CDKN1A* and *BBC3* and IFN pathway genes including *IL2RB* and *IFIT2* were highly upregulated by ATO in R282W cells, but not in C124R cells (Figure 5C).

We next aimed to determine whether ATO can elicit broad-spectrum activation of the IFN immune response in human cancer cells harboring ATO-rescuable mutations apart from R282W. To accomplish this, we reanalyzed the transcriptomes of U937 cell lines harboring 10 type-1 ATO-rescuable human p53 mutations, both with and without ATO treatment, as previously reported[21] (Table S6). Type-1 p53 mutants, despite lower prevalences than one of the hotspot mutants, can be restored with p53 activities comparable to the wild type.[21] GSEA analysis indicated that the ATO-induced changes in the transcriptomes were highly enriched in the p53 pathway, IFN-γ response, and IFN-α response, as well as "apoptosis" and "TNF-α signaling via NF-κB," which are closely related to p53 and immune/inflammation-related pathways, respectively (Figure 5D shows pooled samples results; Figure S5A show the individual sample results). Among the DEGs (FC $\geq$ 2 or $\leq$ 0.5, $p < 0.05$) upon ATO treatment, a set of upregulated p53 targets and IFN pathway genes were observed (Figures 5E and S5B). Heatmaps revealed that ATO treatment resulted in the global upregulation of p53 targets and IFN pathway genes in these ten cell lines (Figure 5F).

We previously conducted a series of cancer patient-derived xenograft (PDX) studies, during which we observed that ATO could effectively rescue p53-R282W (based on the observed

pathways (IFN-α/γ response) are shown as purple bars. (B) Volcano plot showing DEGs (FC $\geq$ 2 or $\leq$0.5, GFOLD $\geq$ 1 or ≤ -1, FPKM $\geq$ 1) upon ATO treatment in R282W and C124R cells. Differentially expressed p53 targets and IFN pathway genes are depicted in red and purple, respectively. (C) Heatmaps showing mRNA expression of established p53 targets (left) and IFN pathway genes (right) in the indicated cells. The mRNA levels of each gene were normalized to the mean expression of the gene in all samples.

(D–F) Transcriptome analysis of U937 cells expressing type-1 p53 mutants upon ATO treatment. The data were compiled from GSE182565. (D) Top five enriched pathways upon ATO treatment as in (A). (E) Volcano plot showing DEGs upon ATO treatment as in (B). (F) Heatmaps showing the mRNA expression of the indicated genes as in (C).

(G and H) Patient-derived xenograft (PDX) tumor tissues of CR2258 model and PA1233 model harboring R282W mutation treated with or without ATO, followed by RNA-seq analysis (n = 3 mice per group). (G) Heatmaps showing the mRNA expression of the established p53 targets in the indicated two PDX models. (H) Heatmaps showing the mRNA expression of the IFN pathway genes in the indicated two PDX models.

(I) Immunoblotting of ISG15 and IFIT1 in the indicated two PDX model tumors from the PBS and ATO groups (n = 5 mice per group).

(J–M) scRNA-seq of PBMCs harvested from a patient at diagnosis and after one cycle of ATO treatment. The data were compiled from GSE182926. (J) t-SNE plot depicting seven classified cell populations. The samples of at-diagnosis and ATO-treated were integrated with two healthy samples (derived from 10k Human PBMCs Multiplexed datasets) to classify cell populations. (K) The top five pathways enriched in the hallmark gene sets for the DEGs upregulated upon ATO treatment in the defined cancer cells. (L) Volcano plot showing DEGs (FC $\geq$ 1.5 or $\leq$0.67, $p < 10^{-20}$) upon ATO treatment in the defined cancer cells. (M) Dot plot showing the expression of the indicated interactions of ligand-receptor pairs between cancer cells and T cells before (at-diagnosis) and after (treated) ATO treatment. p values are indicated by circle size (permutation test). The means of the average expression levels of the interactions are indicated in color.

protein-level upregulation of p53 targets) and inhibited human colon and pancreas PDX models (CR2258 and PA1233 models).[34] Tumor tissues from each of the PBS and ATO groups in CR2258 and PA1233 models were thus collected and subjected to RNA-seq (Table S7). Heatmaps of p53 target genes showed that ATO treatment globally upregulated p53 targets at the mRNA level, confirming our previously reported upregulation at the protein level (Figure 5G). Heatmaps of IFN pathway genes suggest that ATO also triggered non-cell-autonomous activation of IFN response (Figure 5H). We examined the changes in protein levels of ISG15 and IFIT1 and found that their expression was elevated in the ATO-treated group compared with the control group (Figure 5I).

We previously reported the first-in-human mutant p53 reactivation in an ATO-treated leukemia patient with a type-1 V272M mutation.[21] By reanalyzing single-cell RNA-seq (scRNA-seq) data from peripheral blood mononuclear cells (PBMCs) derived from this patient before (termed at-diagnosis) and after (termed treated) ATO treatment (GSE182926), we identified 19 cell clusters by mapping with healthy samples through unsupervised clustering in a t-distributed stochastic neighbor embedding (t-SNE) plot (Figure S5C). The two samples and healthy controls were separated, and seven cell types, including cancer cells, erythroblasts, and five types of immune cells, were distinguished using marker genes (Figures 5J and S5D). Pathway analysis of the upregulated DEGs in the defined cancer cells (treated vs. at-diagnosis; FC $\geq$ 1.5 or $\leq$0.67, $p < 10^{-20}$) revealed strong enrichment in the "p53 pathway," as well as the "IFN-γ response" and several other immune/inflammation-related pathways in the MSigDB hallmark gene sets (Figure 5K). Among the DEGs, 14 p53 targets, including *CDKN1A* and *TNFRSF10D*, and 11 IFN genes, including *IFIT2*, were significantly upregulated after ATO therapy (Figure 5L; examples seen in Figure S5E). Interactions between cells play a critical role in the immune response to tumorigenesis and progression.[32] Systematic CellPhoneDB analysis[35] suggested that ATO treatment triggered more active cell-cell interactions between cancer cells and the five major immune cells (Figure S5F). Regarding the key interaction between T cells and cancer cells, increased expression of ligand-receptor pairs *MIF_TNFRSF10D*, *CD55_ADGRE5*, *CD226_NECTIN2*, *CCL3L1_CCR1*, and *CCL3_CCR1* was observed (Figure 5M). Interestingly, *TNFRSF10D* is a well-established p53 target.[23] These in-patient findings are consistent with the earlier observations of T cell infiltration in tumors from ATO-treated mice.

Wild-type p53-associated immune signatures across cancer types

Due to the relatively low frequency of p53 mutations in acute promyelocytic leukemia (APL, the leukemia subtype approved for ATO treatment) and non-APL leukemia, as well as the limited number of the currently reported ATO-treated solid-tumor patients, we failed to identify ATO-treated patients with known p53 mutational status, except the patient[21] that has been analyzed in Figures 5J–5M. To investigate the relationship between the immune microenvironment and p53 function, we analyzed data from cancer patients enrolled in The Cancer Genome Atlas (TCGA) Pan-Cancer Atlas dataset, comprising 9,875 individuals across 33 cancer types (Figure 6A). We specifically focused on the 20 cancer types with a p53 mutation rate exceeding 5% and a sample size of over 100 patients (Figure 6A), conducting a thorough examination of their immune landscape.

Patients were divided into p53-wild-type (p53-wt) and p53-mutant (p53-mut) groups, representing functional p53 (akin to ATO-rescued mutant p53) and non-functional p53 (mutant p53 without ATO treatment), respectively. Initially, we calculated immune scores using the estimate algorithm. The immune score of acute myeloid leukemia (LAML) was also calculated since differentiation of the major immune cells (T, B, NK cells, etc.) in this disease is largely normal. It indicated that the level of immune response of p53-wt samples was significantly higher than that of p53-mut samples in 6 cancer types (lung squamous cell carcinoma [LUSC], stomach adenocarcinoma [STAD], head and neck squamous cell carcinoma [HNSC], cervical squamous cell carcinoma and endocervical adenocarcinoma [CESC], uterine corpus endometrial carcinoma [UCEC], and colon adenocarcinoma [COAD]), while only 2 cancer types had significantly lower levels of immune response in p53-wt samples (Figure 6B, red dots and blue dots, respectively). Furthermore, by analyzing gene signatures from tumor-infiltrating T cells across the Pan-Cancer cohorts,[36] we calculated the T cell abundance score for various cancer types. This analysis revealed that the abundance of both $CD4^+$ and $CD8^+$ T cells was overall higher in p53-wt samples across cancer types (Figure 6C, there are more red squares than blue squares). Similarly, by calculating the average gene expression values of the IFN pathway and the chemokine signaling pathway, we found that both pathways were overall more activated in the p53-wt samples (Figure 6D, 5–6 cancer types vs. 2 cancer types). It is notable that, in the aforementioned three assays, the five cancer types—LUSC, HNSC, STAD, COAD, and UCEC—are consistently the cancer types that exhibit a strong correlation between p53-wt state and immune response (Figures 6B–6D). We noticed that CESC was an exceptional cancer type that has higher immune score; however, it did not show higher expression of genes in the IFN α/γ pathway or chemokine signaling pathway in p53-wt patients as compared to p53-mut patients (Figure 6B compared to Figure 6D). This may be due to the high prevalence of human papillomavirus in CESC,[37] wherein the E6 proteins target wild-type p53 for proteasomal degradation.[38] Breast invasive carcinoma is the only cancer type that consistently suggests poorer immune response in p53-wt samples, and the underlying mechanism remains to be further studied.

DISCUSSION

Here we reported that pharmacological rescue of the hotspot R279W could effectively prolong mouse survival and, encouragingly, involve regression of spontaneous lymphoma (Figure 7, upper panel). However, ATO did not always potently trigger tumor regression among mice, despite the consistently observed mutant p53 reactivation. The inconsistency is potentially caused by the complex and multifaceted nature of the immune system in living animals. This may also be attributed to the inherent individual variation among the mice, particularly in the current long-duration living animal studies.

Our mechanistic studies revealed that the activation of $CD8^+$ T cells following immune response was partly indispensable in

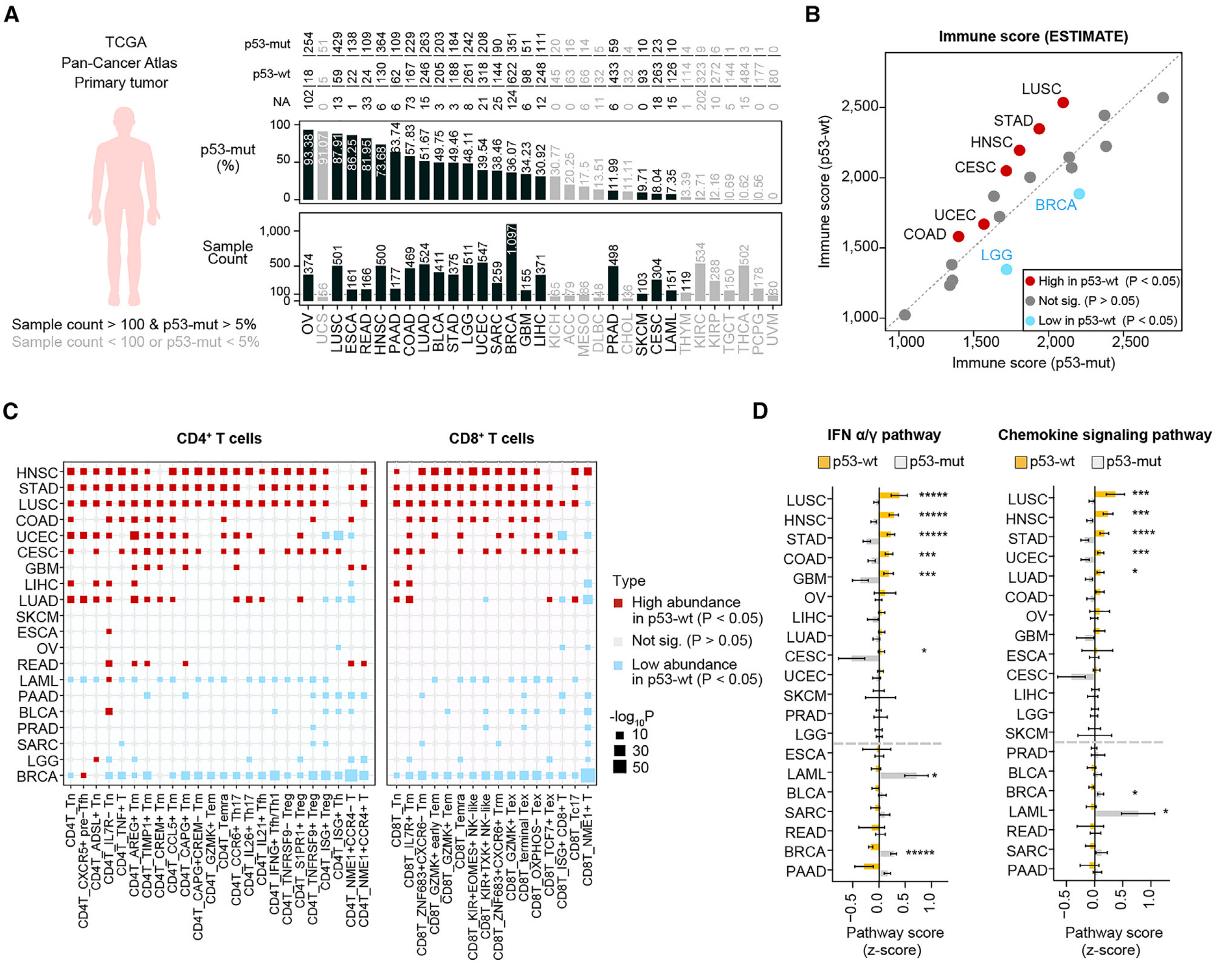

Figure 6. Wild-type p53-associated immune signatures across cancer types

(A) Overview of sample information and p53 mutation frequencies in the TCGA PanCanAtlas. 9,875 primary tumor samples spanning 33 different cancer types were illustrated, with sample size and p53 mutated frequencies of each cancer type presented. Cancers with a sample size of more than 100 patients and a p53 mutation rate of more than 5% are labeled black for subsequent analysis (20 cancer types included). Others are marked in gray.

(B) Immune scores of p53-wild-type (p53-wt) and p53 mutated (p53-mut) samples of TCGA PanCanAtlas. Each dot on the plot represents an individual TCGA cohort, with the mean immune score calculated for visual representation.

(C) Abundance score of tumor-infiltrating T cells in TCGA cancer types. Red dots indicate the significantly higher abundance of tumor-infiltrating T cells in p53-wt samples compared to p53-mut samples ($p < 0.05$), while blue dots represent significantly lower abundance ($p < 0.05$).

(D) Bar plot of mean gene expression of IFN pathway and chemokine signaling pathway. p values were calculated using the two-sided Whitney U test; *$p < 0.05$. Full name of the aberrations of the 33 TCGA Pan-Cancer cohorts can be found in https://gdc.cancer.gov/about-data/publications/pancanatlas.

the ATO-triggered tumor regression (Figure 7, middle panel). The exact signaling underlying how the rescued p53 mutant triggers anticancer immune responses requires further investigation. Cell senescence is reported to trigger antitumor immune responses through the axis of p21-Rb-chemokine-T cells,[39] involving activation of DCs and CD8+ T cells.[40] Interestingly, wild-type p53 reintroduction into engineered p53-deficient mice, reminiscent of pharmacological reactivation of mutant p53, can induce senescence and trigger antitumor immunity,[10,11] defining the unconventional non-cell-autonomous tumor suppression function of p53.[41] Apart from *CDKN1A*, other p53 targets may also engage in the observed immune response triggered by the rescued mutant p53. For example, many key immune-regulating genes, such as *CXCR2*,[42] *IRF5*,[25] *IRF9*,[42] *ISG15*,[27] *TLR3*,[43] and *TNFRSF10D*,[23] have been reported as direct targets of p53. It is worth noting that ATO also exhibits anticancer efficacy in (partly) immunodeficient mice in the current models (Figures 4I, 4M, and 4P), consistent with our previous mouse studies.[18,33] This should be attributed to the classic cell-autonomous functions of p53 upon ATO treatment, such as cell-cycle arrest and pro-apoptotic functions (Figures 4R–4T). Together, the observed tumor suppression ability of ATO in immunocompetent mice can be attributed to both the well-established classic cell-autonomous functions and currently observed non-cell-autonomous

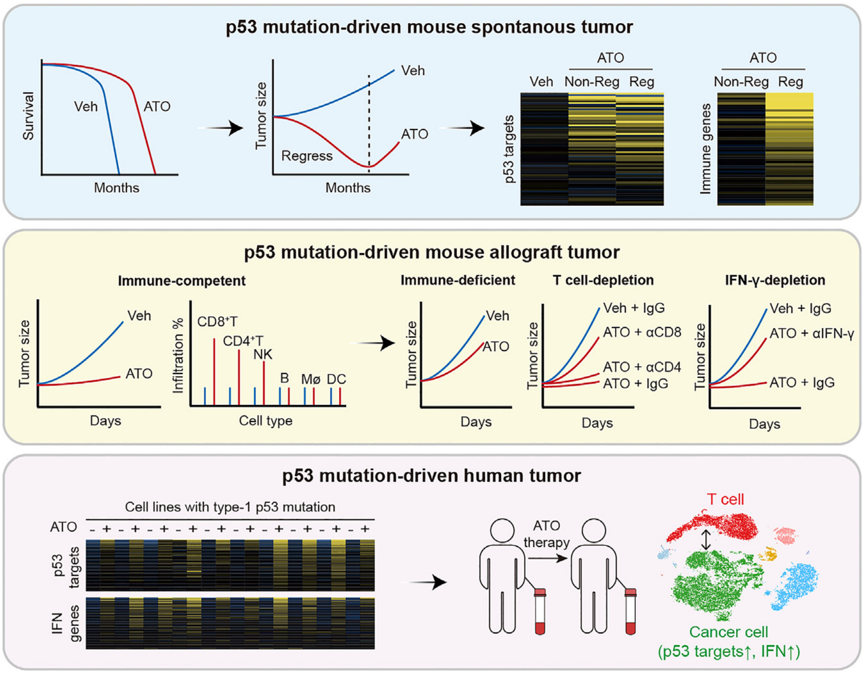

Figure 7. Summary of the study

Upper, pharmacologically rescued p53-R279W extends W/W mice survival and triggers regression of spontaneous tumors, accompanied by p53 reactivation and immune response. Middle, $CD8^+$ T cells and IFN-γ are largely indispensable in the tumor suppression action of ATO-rescued p53-R279W in mice transplanted with W/W lymphoma cells. Lower, ATO broad-spectrum upregulated p53 targets and IFN pathway genes in cell lines harboring 10 type-1 human p53 mutants. ATO therapy upregulated p53 targets and IFN pathway genes and promoted T cell-cancer cell interaction in a leukemia patient harboring the type-1 V272M mutant.

immune activation functions. The contribution of these two "arms" of ATO may probably vary depending on the tumor type, tumor microenvironment, and immuno-competence of the host.

It is important to note that the immune response can be elicited broadly by ATO-rescued type-1 p53 mutants in tissue culture and in an ATO-treated patient (Figure 7, lower panel). Together, TSGs could potentially serve as alternative targets to the limited immune-modulating genes established thus far for anticancer immune therapy.[44]

Limitations of the study

Our study highlights the potential of pharmacological rescue of mutant p53 in triggering anticancer immune response by using the mouse model harboring spontaneous tumors, but several limitations exist. A major limitation is that the exact signaling underlying how the rescued p53 mutant triggers anticancer immune responses requires further exploration. In addition, translational challenges remain, as results from animal models may not fully mimic the complexity of human cancers, highlighting the need for further studies to validate these findings in clinical settings.

RESOURCE AVAILABILITY

Lead contact

Further information and requests for resources and reagents should be directed to and will be fulfilled by the lead contact, Min Lu (min.lu@shsmu.edu.cn).

Materials availability

This study did not generate new unique reagents.

Data and code availability

- This study's raw and processed RNA-seq data are available at the Gene Expression Omnibus (GEO, RRID: SCR_005012) under the accession number GEO: GSE229093.
- The code used for the data analysis in this paper is available on GitLab: https://nrctm-bioinfo.github.io/p53_immune_2025 and has been deposited at Zenodo: https://doi.org/10.5281/zenodo.14649087; DOI is listed in the key resources table.
- Any additional information required to reanalyze the data reported in this work paper is available from the lead contact (min.lu@shsmu.edu.cn) upon request.

ACKNOWLEDGMENTS

We thank the BL17U/BL18U1/BL19U1/BL19U2/BL01B beamlines of the National Center for Protein Science Shanghai (NCPSS) at Shanghai Synchrotron Radiation Facility (SSRF) for the analysis of the structure of p53 ABP pocket. This work was funded by the Noncommunicable Chronic Diseases-National Science and Technology Major Project (2023 ZD0501300 and 2024ZD0519600, M.L.), National Natural Science Foundation of China (82425001 and 82130075, M.L.; 82301637, B.W.;82403726, H.S.; 823B2004, X.C.; 82202872, Jiale Wu), SJTU Trans-med Awards Research (20210104, M.L.), Dawn Program of Shanghai Education Commission (21SG18, M.L.), and Collaborative Innovation Center for Clinical and Translational Science by Ministry of Education & Shanghai (CCTS-202401, M.L.).

AUTHOR CONTRIBUTIONS

M.L., Sujiang Zhang, H.S., and X.D. conceived the study and designed the experiments. J.L. performed most of the experiments and analyzed the data. Shuang Zhang. performed the proteomics analyses. Y.D. analyzed clinical patient data. D.L. helped analyze the data and performed the animal experiments. B.W., Jiale Wu, Y.L., S.X., Z.W., Jiaqi Wu, D.Z., X.C., K.T., and F.S. constructed plasmids and performed cell experiments. M.L. and J.L. wrote the draft manuscript.

DECLARATION OF INTERESTS

The authors declare no competing interests.

STAR★METHODS

Detailed methods are provided in the online version of this paper and include the following:

- KEY RESOURCES TABLE
- EXPERIMENTAL MODEL AND STUDY PARTICIPANT DETAILS
  - p53-R279W knockin mice and the animals used
  - Cell lines and cell culture
  - Survival study of W/W mice
  - Isolation of primary tumor cells
- METHOD DETAILS
  - Luciferase reporter assay
  - Immunohistochemistry and immunoblotting
  - Quantitative PCR
  - High-resolution ultrasound imaging and analysis
  - Allograft model study
  - RNA-sequencing
  - Flow cytometric analysis
  - ScRNA-seq of patient's PBMCs
  - Proteomics profiling
  - TCGA Pan-Cancer Atlas data analysis
- QUANTIFICATION AND STATISTICAL ANALYSIS

SUPPLEMENTAL INFORMATION

Supplemental information can be found online at https://doi.org/10.1016/j.xcrm.2025.101976.

Received: June 12, 2023
Revised: August 5, 2024
Accepted: January 28, 2025
Published: February 21, 2025

REFERENCES

1. Martinez-Jimenez, F., Muinos, F., Sentis, I., Deu-Pons, J., Reyes-Salazar, I., Arnedo-Pac, C., Mularoni, L., Pich, O., Bonet, J., Kranas, H., et al. (2020). A compendium of mutational cancer driver genes. Nat. Rev. Cancer *20*, 555–572. https://doi.org/10.1038/s41568-020-0290-x.

2. Chakravarty, D., Gao, J., Phillips, S.M., Kundra, R., Zhang, H., Wang, J., Rudolph, J.E., Yaeger, R., Soumerai, T., Nissan, M.H., et al. (2017). OncoKB: A precision oncology knowledge base. JCO Precision Oncology. JCO Precis. Oncol. *2017*, 1–16. https://doi.org/10.1200/PO.17.00011.

3. Martin, T.D., Patel, R.S., Cook, D.R., Choi, M.Y., Patil, A., Liang, A.C., Li, M.Z., Haigis, K.M., and Elledge, S.J. (2021). The adaptive immune system is a major driver of selection for tumor suppressor gene inactivation. Science *373*, 1327–1335. https://doi.org/10.1126/science.abg5784.

4. Lawson, K.A., Sousa, C.M., Zhang, X., Kim, E., Akthar, R., Caumanns, J.J., Yao, Y., Mikolajewicz, N., Ross, C., Brown, K.R., et al. (2020). Functional genomic landscape of cancer-intrinsic evasion of killing by T cells. Nature *586*, 120–126. https://doi.org/10.1038/s41586-020-2746-2.

5. Donehower, L.A., Harvey, M., Slagle, B.L., McArthur, M.J., Montgomery, C.A., Jr., Butel, J.S., and Bradley, A. (1992). Mice deficient for p53 are developmentally normal but susceptible to spontaneous tumours. Nature *356*, 215–221. https://doi.org/10.1038/356215a0.

6. Feldser, D.M., Kostova, K.K., Winslow, M.M., Taylor, S.E., Cashman, C., Whittaker, C.A., Sanchez-Rivera, F.J., Resnick, R., Bronson, R., Hemann, M.T., and Jacks, T. (2010). Stage-specific sensitivity to p53 restoration during lung cancer progression. Nature *468*, 572–575. https://doi.org/10.1038/nature09535.

7. Junttila, M.R., Karnezis, A.N., Garcia, D., Madriles, F., Kortlever, R.M., Rostker, F., Brown Swigart, L., Pham, D.M., Seo, Y., Evan, G.I., and Martins, C.P. (2010). Selective activation of p53-mediated tumour suppression in high-grade tumours. Nature *468*, 567–571. https://doi.org/10.1038/nature09526.

8. Martins, C.P., Brown-Swigart, L., and Evan, G.I. (2006). Modeling the therapeutic efficacy of p53 restoration in tumors. Cell *127*, 1323–1334. https://doi.org/10.1016/j.cell.2006.12.007.

9. Ventura, A., Kirsch, D.G., McLaughlin, M.E., Tuveson, D.A., Grimm, J., Lintault, L., Newman, J., Reczek, E.E., Weissleder, R., and Jacks, T. (2007). Restoration of p53 function leads to tumour regression in vivo. Nature *445*, 661–665. https://doi.org/10.1038/nature05541.

10. Xue, W., Zender, L., Miething, C., Dickins, R.A., Hernando, E., Krizhanovsky, V., Cordon-Cardo, C., and Lowe, S.W. (2007). Senescence and tumour clearance is triggered by p53 restoration in murine liver carcinomas. Nature *445*, 656–660. https://doi.org/10.1038/nature05529.

11. Chen, H.A., Ho, Y.J., Mezzadra, R., Adrover, J.M., Smolkin, R., Zhu, C., Woess, K., Bernstein, N., Schmitt, G., Fong, L., et al. (2023). Senescence Rewires Microenvironment Sensing to Facilitate Antitumor Immunity. Cancer Discov. *13*, 432–453. https://doi.org/10.1158/2159-8290.CD-22-0528.

12. Valente, L.J., Gray, D.H.D., Michalak, E.M., Pinon-Hofbauer, J., Egle, A., Scott, C.L., Janic, A., and Strasser, A. (2013). p53 efficiently suppresses tumor development in the complete absence of its cell-cycle inhibitory and proapoptotic effectors p21, Puma, and Noxa. Cell Rep. *3*, 1339–1345. https://doi.org/10.1016/j.celrep.2013.04.012.

13. Li, T., Kon, N., Jiang, L., Tan, M., Ludwig, T., Zhao, Y., Baer, R., and Gu, W. (2012). Tumor suppression in the absence of p53-mediated cell-cycle arrest, apoptosis, and senescence. Cell *149*, 1269–1283. https://doi.org/10.1016/j.cell.2012.04.026.

14. Brady, C.A., Jiang, D., Mello, S.S., Johnson, T.M., Jarvis, L.A., Kozak, M.M., Kenzelmann Broz, D., Basak, S., Park, E.J., McLaughlin, M.E., et al. (2011). Distinct p53 transcriptional programs dictate acute DNA-damage responses and tumor suppression. Cell *145*, 571–583. https://doi.org/10.1016/j.cell.2011.03.035.

15. Marin, I., Boix, O., Garcia-Garijo, A., Sirois, I., Caballe, A., Zarzuela, E., Ruano, I., Attolini, C.S.O., Prats, N., López-Domínguez, J.A., et al. (2023). Cellular Senescence Is Immunogenic and Promotes Antitumor Immunity. Cancer Discov. *13*, 410–431. https://doi.org/10.1158/2159-8290.Cd-22-0523.

16. Sabapathy, K., and Lane, D.P. (2018). Therapeutic targeting of p53: all mutants are equal, but some mutants are more equal than others. Nat. Rev. Clin. Oncol. *15*, 13–30. https://doi.org/10.1038/nrclinonc.2017.151.

17. Hassin, O., and Oren, M. (2023). Drugging p53 in cancer: one protein, many targets. Nat. Rev. Drug Discov. *22*, 127–144. https://doi.org/10.1038/s41573-022-00571-8.

18. Chen, S., Wu, J.L., Liang, Y., Tang, Y.G., Song, H.X., Wu, L.L., Xing, Y.F., Yan, N., Li, Y.T., Wang, Z.Y., et al. (2021). Arsenic Trioxide Rescues Structural p53 Mutations through a Cryptic Allosteric Site. Cancer Cell *39*, 225–239.e8. https://doi.org/10.1016/j.ccell.2020.11.013.

19. Xiao, S., Shi, F., Song, H., Cui, J., Zheng, D., Zhang, H., Tan, K., Wu, J., Chen, X., Wu, J., et al. (2024). Characterization of the generic mutant p53-rescue compounds in a broad range of assays. Cancer Cell *42*, 325–327. https://doi.org/10.1016/j.ccell.2024.01.008.

20. Song, H., Xiao, S., Wu, J., and Lu, M. (2024). Drugging p53: Barriers, Criteria, and Prospects. Cancer Discov. *14*, 2055–2060. https://doi.org/10.1158/2159-8290.CD-24-0837.

21. Song, H., Wu, J., Tang, Y., Dai, Y., Xiang, X., Li, Y., Wu, L., Wu, J., Liang, Y., Xing, Y., et al. (2023). Diverse rescue potencies of p53 mutations to ATO are predetermined by intrinsic mutational properties. Sci. Transl. Med. *15*, eabn9155. https://doi.org/10.1126/scitranslmed.abn9155.

22. Bachy, E., Urb, M., Chandra, S., Robinot, R., Bricard, G., de Bernard, S., Traverse-Glehen, A., Gazzo, S., Blond, O., Khurana, A., et al. (2016). CD1d-restricted peripheral T cell lymphoma in mice and humans. J. Exp. Med. *213*, 841–857. https://doi.org/10.1084/jem.20150794.

23. Fischer, M. (2017). Census and evaluation of p53 target genes. Oncogene *36*, 3943–3956. https://doi.org/10.1038/onc.2016.502.

CellPress OPEN ACCESS

24. Lazear, H.M., Schoggins, J.W., and Diamond, M.S. (2019). Shared and Distinct Functions of Type I and Type III Interferons. Immunity *50*, 907–923. https://doi.org/10.1016/j.immuni.2019.03.025.

25. Mori, T., Anazawa, Y., Iiizumi, M., Fukuda, S., Nakamura, Y., and Arakawa, H. (2002). Identification of the interferon regulatory factor 5 gene (IRF-5) as a direct target for p53. Oncogene *21*, 2914–2918. https://doi.org/10.1038/sj.onc.1205459.

26. Muñoz-Fontela, C., Macip, S., Martinez-Sobrido, L., Brown, L., Ashour, J., Garcia-Sastre, A., Lee, S.W., and Aaronson, S.A. (2008). Transcriptional role of p53 in interferon-mediated antiviral immunity. J. Exp. Med. *205*, 1929–1938. https://doi.org/10.1084/jem.20080383.

27. Hummer, B.T., Li, X.L., and Hassel, B.A. (2001). Role for p53 in gene induction by double-stranded RNA. J. Virol. *75*, 7774–7777. https://doi.org/10.1128/JVI.75.16.7774-7777.2001.

28. Balkwill, F. (2004). Cancer and the chemokine network. Nat. Rev. Cancer *4*, 540–550. https://doi.org/10.1038/nrc1388.

29. Chen, H., Cong, X., Wu, C., Wu, X., Wang, J., Mao, K., Li, J., Zhu, G., Liu, F., Meng, X., et al. (2020). Intratumoral delivery of CCL25 enhances immunotherapy against triple-negative breast cancer by recruiting CCR9+ T cells. Sci. Adv. *6*, eaax4690. https://doi.org/10.1126/sciadv.aax4690.

30. Li, H., Chen, X., Zeng, W., Zhou, W., Zhou, Q., Wang, Z., Jiang, W., Xie, B., and Sun, L.Q. (2020). Radiation-enhanced expression of CCL22 in nasopharyngeal carcinoma is associated with CCR4+ CD8 T cell recruitment. Int. J. Radiat. Oncol. Biol. Phys. *108*, 126–139. https://doi.org/10.1016/j.ijrobp.2020.05.001.

31. Jie, X., Chen, Y., Zhao, Y., Yang, X., Xu, Y., Wang, J., Meng, R., Zhang, S., Dong, X., Zhang, T., et al. (2022). Targeting KDM4C enhances CD8+ T cell mediated antitumor immunity by activating chemokine CXCL10 transcription in lung cancer. J. Immunother. Cancer *10*, e003716. https://doi.org/10.1136/jitc-2021-003716.

32. Binnewies, M., Roberts, E.W., Kersten, K., Chan, V., Fearon, D.F., Merad, M., Coussens, L.M., Gabrilovich, D.I., Ostrand-Rosenberg, S., Hedrick, C.C., et al. (2018). Understanding the tumor immune microenvironment (TIME) for effective therapy. Nat. Med. *24*, 541–550. https://doi.org/10.1038/s41591-018-0014-x.

33. Song, H., Wu, J., Tang, Y., Dai, Y., Xiang, X., Li, Y., Wu, L., Wu, J., Liang, Y., Xing, Y., et al. (2023). Diverse rescue potencies of p53 mutations to ATO are predetermined by intrinsic mutational properties. Sci. Transl. Med. *15*, eabn9155. https://doi.org/10.1126/scitranslmed.abn9155.

34. Song, H., Wu, J., Tang, Y., Dai, Y., Xiang, X., Li, Y., Wu, L., Wu, J., Liang, Y., Xing, Y., et al. (2023). Diverse rescue potencies of p53 mutations to ATO are predetermined by intrinsic mutational properties. Sci. Transl. Med. *15*, eabn9155. https://doi.org/10.1126/scitranslmed.abn9155.

35. Cang, Z., Zhao, Y., Almet, A.A., Stabell, A., Ramos, R., Plikus, M.V., Atwood, S.X., and Nie, Q. (2023). Screening cell-cell communication in spatial transcriptomics via collective optimal transport. Nat. Methods *20*, 218–228. https://doi.org/10.1038/s41592-022-01728-4.

36. Zheng, L., Qin, S., Si, W., Wang, A., Xing, B., Gao, R., Ren, X., Wang, L., Wu, X., Zhang, J., et al. (2021). Pan-cancer single-cell landscape of tumor-infiltrating T cells. Science *374*, abe6474. https://doi.org/10.1126/science.abe6474.

37. Warburton, A., Markowitz, T.E., Katz, J.P., Pipas, J.M., and McBride, A.A. (2021). Recurrent integration of human papillomavirus genomes at transcriptional regulatory hubs. NPJ Genom. Med. *6*, 101. https://doi.org/10.1038/s41525-021-00264-y.

38. Werness, B.A., Levine, A.J., and Howley, P.M. (1990). Association of human papillomavirus types 16 and 18 E6 proteins with p53. Science *248*, 76–79.

39. Sturmlechner, I., Zhang, C., Sine, C.C., van Deursen, E.J., Jeganathan, K.B., Hamada, N., Grasic, J., Friedman, D., Stutchman, J.T., Can, I., et al. (2021). p21 produces a bioactive secretome that places stressed cells under immunosurveillance. Science *374*, eabb3420. https://doi.org/10.1126/science.abb3420.

40. Marin, I., Boix, O., Garcia-Garijo, A., Sirois, I., Caballe, A., Zarzuela, E., Ruano, I., Attolini, C.S.O., Prats, N., López-Domínguez, J.A., et al. (2023). Cellular senescence is immunogenic and promotes anti-tumor immunity. Cancer Discov. *13*, 410–431. https://doi.org/10.1158/2159-8290.CD-22-0523.

41. Lujambio, A., Akkari, L., Simon, J., Grace, D., Tschaharganeh, D.F., Bolden, J.E., Zhao, Z., Thapar, V., Joyce, J.A., Krizhanovsky, V., and Lowe, S.W. (2013). Non-cell-autonomous tumor suppression by p53. Cell *153*, 449–460. https://doi.org/10.1016/j.cell.2013.03.020.

42. Guo, H., Liu, Z., Xu, B., Hu, H., Wei, Z., Liu, Q., Zhang, X., Ding, X., Wang, Y., Zhao, M., et al. (2013). Chemokine receptor CXCR2 is transactivated by p53 and induces p38-mediated cellular senescence in response to DNA damage. Aging Cell *12*, 1110–1121. https://doi.org/10.1111/acel.12138.

43. Taura, M., Eguma, A., Suico, M.A., Shuto, T., Koga, T., Komatsu, K., Komune, T., Sato, T., Saya, H., Li, J.D., and Kai, H. (2008). p53 regulates Toll-like receptor 3 expression and function in human epithelial cell lines. Mol. Cell Biol. *28*, 6557–6567. https://doi.org/10.1128/MCB.01202-08.

44. Wu, J., Song, H., Wang, Z., and Lu, M. (2021). Three optimized assays for the evaluation of compounds that can rescue p53 mutants. STAR Protoc. *2*, 100688. https://doi.org/10.1016/j.xpro.2021.100688.

45. Martin, M. (2011). Cutadapt removes adapter sequences from high-throughput sequencing reads. EMBnet. j. *17*, 10–12. Embnet Journal *17*.

46. Kim, D., Paggi, J.M., Park, C., Bennett, C., and Salzberg, S.L. (2019). Graph-based genome alignment and genotyping with HISAT2 and HISAT-genotype. Nat. Biotechnol. *37*, 907–915.

47. Anders, S., Pyl, P.T., and Huber, W. (2015). HTSeq—a Python framework to work with high-throughput sequencing data. Bioinformatics, 166–169.

48. Robinson, M.D., Mccarthy, D.J., and Smyth, G.K. (2010). EdgeR: a Bioconductor package for differential expression analysis of digital gene expression data. Bioinformatics *26*, 139–140.

49. Erkek, S., Johann, P., Kerl, K., Finetti, M., Zapatka, M., Fruehwald, M.C., Chavez, L., Gajjar, A., Williamson, D., Hasselblatt, M., et al. (2016). GENT-31. PEDcBIOPORTAL: A CANCER DATA VISUALIZATION TOOL FOR INTEGRATIVE PEDIATRIC CANCER ANALYSES. Neuro Oncol., vi80.

50. Feng, J., Meyer, C.A., Wang, Q., Liu, J.S., Shirley Liu, X., and Zhang, Y. (2012). GFOLD: a generalized fold change for ranking differentially expressed genes from RNA-seq data. Bioinformatics *28*, 2782–2788.

51. Wisniewski, J.R., Zougman, A., Nagaraj, N., and Mann, M. (2009). Universal sample preparation method for proteome analysis. Nat. Methods *6*, 359–U360. https://doi.org/10.1038/nmeth.1322.

52. Demichev, V., Messner, C.B., Vernardis, S.I., Lilley, K.S., and Ralser, M. (2020). DIA-NN: neural networks and interference correction enable deep proteome coverage in high throughput. Nat. Methods *17*, 41–44. https://doi.org/10.1038/s41592-019-0638-x.

53. Wang, S., Xiong, Y., Zhao, L., Gu, K., Li, Y., Zhao, F., Li, J., Wang, M., Wang, H., Tao, Z., et al. (2022). UCSCXenaShiny: an R/CRAN package for interactive analysis of UCSC Xena data. Bioinformatics *38*, 527–529. https://doi.org/10.1093/bioinformatics/btab561.

54. Fan, Y., Xi, L., Hughes, D.S.T., Zhang, J., Zhang, J., Futreal, P.A., Wheeler, D.A., and Wang, W. (2016). MuSE: accounting for tumor heterogeneity using a sample-specific error model improves sensitivity and specificity in mutation calling from sequencing data. Genome Biol. *17*, 178. https://doi.org/10.1186/s13059-016-1029-6.

55. Ellrott, K., Bailey, M.H., Saksena, G., Covington, K.R., Kandoth, C., Stewart, C., Hess, J., Ma, S., Chiotti, K.E., McLellan, M., et al. (2018). Scalable Open Science Approach for Mutation Calling of Tumor Exomes Using Multiple Genomic Pipelines. Cell Syst. *6*, 271–281.e7. https://doi.org/10.1016/j.cels.2018.03.002.

56. Ellrott, K., Bailey, M.H., Saksena, G., Covington, K.R., Kandoth, C., Stewart, C., Hess, J., Ma, S., Chiotti, K.E., McLellan, M., et al. (2018). Scalable Open Science Approach for Mutation Calling of Tumor Exomes Using Multiple Genomic Pipelines. Cell Syst. *6*, 271–281.e7. https://doi.org/10.1016/j.cels.2018.03.002.

57. Cibulskis, K., Lawrence, M.S., Carter, S.L., Sivachenko, A., Jaffe, D., Sougnez, C., Gabriel, S., Meyerson, M., Lander, E.S., and Getz, G. (2013). Sensitive detection of somatic point mutations in impure and heterogeneous cancer samples. Nat. Biotechnol. *31*, 213–219. https://doi.org/10.1038/nbt.2514.

58. Larson, D.E., Harris, C.C., Chen, K., Koboldt, D.C., Abbott, T.E., Dooling, D.J., Ley, T.J., Mardis, E.R., Wilson, R.K., and Ding, L. (2012). SomaticSniper: identification of somatic point mutations in whole genome sequencing data. Bioinformatics *28*, 311–317. https://doi.org/10.1093/bioinformatics/btr665.

59. Koboldt, D.C., Zhang, Q., Larson, D.E., Shen, D., McLellan, M.D., Lin, L., Miller, C.A., Mardis, E.R., Ding, L., and Wilson, R.K. (2012). VarScan 2: somatic mutation and copy number alteration discovery in cancer by exome sequencing. Genome Res. *22*, 568–576. https://doi.org/10.1101/gr.129684.111.

60. Yoshihara, K., Shahmoradgoli, M., Martínez, E., Vegesna, R., Kim, H., Torres-Garcia, W., Treviño, V., Shen, H., Laird, P.W., Levine, D.A., et al. (2013). Inferring tumour purity and stromal and immune cell admixture from expression data. Nat. Commun. *4*, 2612. https://doi.org/10.1038/ncomms3612.

61. Hänzelmann, S., Castelo, R., and Guinney, J. (2013). GSVA: gene set variation analysis for microarray and RNA-seq data. BMC Bioinf. *14*, 1–15. https://doi.org/10.1186/1471-2105-14-7.

STAR★METHODS

KEY RESOURCES TABLE

REAGENT or RESOURCE	SOURCE	IDENTIFIER
Antibodies		
Waf1/Cip1/CDKN1A p21 Antibody (187)	Santa Cruz Biotechnology	Cat# sc-817; RRID: AB_628072
Anti-p53 antibody	Abcam	Cat# ab26; RRID: AB_303198
beta Actin Antibody	Santa Cruz Biotechnology	Cat# sc-47778 HRP; RRID: AB_2714189
Monoclonal Anti-MDM2 antibody	Sigma-Aldrich	Cat# M4308; RRID: AB_260523
ISG15 antibody	Proteintech	Cat# 15981-1-AP; RRID: AB_2126302
IFIT1 antibody	Proteintech	Cat# 23247-1-AP; RRID: AB_2811269
GAPDH Mouse mAb	ABclonal	Cat# AC002; RRID: AB_2736879
Ki67 Rabbit mAb	Beyotime	Cat# AF1738; RRID: AB_3094450
goat anti-rabbit immunoglobulin	Servicebio	Cat# GB23303; RRID: AB_2811189
$CD4^+$ depletion antibody	BioLegend	Cat# 100457; RRID: AB_2810318
$CD8^+$ depletion antibody	BioLegend	Cat# 100763; RRID: AB_2810323
IFN-γ depletion antibody	BioLegend	Cat# 505847; RRID: AB_2616675
CD45.1-FITC antibody	BioLegend	Cat# 110705; RRID: AB_313494
CD3-PE/Cyanine7 antibody	BioLegend	Cat# 100219; RRID: AB_1732068
CD19-Brilliant Violet 510 antibody	BioLegend	Cat# 115545; RRID: AB_2562136
CD4-FITC antibody	BioLegend	Cat# 100405; RRID: AB_312690
CD4-APC/Cyanine7 antibody	BioLegend	Cat# 100413; RRID: AB_312698
CD8a-PE antibody	BioLegend	Cat# 100707; RRID: AB_312746
CD11B-PE/Cyanine7 antibody	BioLegend	Cat# 101215; RRID: AB_312798
CD11C-PerCP antibody	BioLegend	Cat# 117325; RRID: AB_893236
F4/80-Brilliant Violet 510 antibody	BioLegend	Cat# 123135; RRID: AB_2562622
NK1.1-APC antibody	BioLegend	Cat# 156505; RRID: AB_2876525
Bacterial and virus strains		
Escherichia coli DH5α	TIANGEN	Cat# CB101-02
Chemicals, peptides, and recombinant proteins		
Arsenic trioxide (ATO)	Sigma	Cat# 202673
100 × L-Glutamine	Sangon	Cat# E607004
DMEM, High Glucose, Pyruvate	Thermo Fisher	Cat# 11995073
BASIC RPMI 1640 Medium, HEPES	Thermo Fisher	Cat# 22400105
Opti-MEM® I Reduced Serum Medium, GlutaMAX^{TM}	Thermo Fisher	Cat# 51985-034
Penicillin-Streptomycin	Thermo Fisher	Cat# 15140-122
Fetal bovine serum	Moregate	Cat# FBSF-500
Critical commercial assays		
Dual Luciferase Reporter Assay Kit	Vazyme	Cat# DL101-01
FuGENE®6 transfection reagent kit	Promega	Cat# E2691
RIPA buffer	Beyotime	Cat# P0013B
TRIzol reagent	Thermo Fisher	Cat# 15596018CN
HiScript® II Q RT SuperMix for qPCR (+ gDNA wiper)	Vazyme	Cat# R223-01
ChamQTM SYBR® qPCR Master Mix	Vazyme	Cat# Q331-02/03
BSA	Yeasen	Cat# 36101ES80
HilyMax transfection reagent kit	Dojindo	Cat# H357

(*Continued on next page*)

Continued		
REAGENT or RESOURCE	SOURCE	IDENTIFIER
Deposited data		
Raw RNA-seq data of mouse tumors	This study	GEO: GSE229093
Raw RNA-seq data of U937 cells expressing type-1 p53 mutants upon ATO treatment	This study	GEO: GSE182565
Raw scRNA-seq data from the clinical trial for ATO	*Song*et al.	GEO: GSE182926
Analysis code and script	This study	https://nrctm-bioinfo.github.io/p53_immune_2025; https://doi.org/10.5281/zenodo.14649087.
Experimental models: Cell lines		
Human: U937	ATCC	CRL-1593.2
Human: H1299	ATCC	CRL-5803
Oligonucleotides		
Cdkn1a_F 5′- CCTGGTGATGTCCGACCTG - 3′	This study	N/A
Cdkn1a_R 5′- CCATGAGCGCATCGCAATC - 3′	This study	N/A
Mdm2_F 5′- GCGTGGAATTTGAAGTTGAGTC - 3′	This study	N/A
Mdm2_R 5′- CTGTATCGCTTTCTCCTGTCTG - 3′	This study	N/A
Bbc3_F 5′- CCTCTGGAAGTGCTGGGAAG - 3′	This study	N/A
Bbc3_R 5′- CTCCCTGGAGCCTATGGGT - 3′	This study	N/A
Bax_F 5′- TTGGAGATGAACTGGACAGC - 3′	This study	N/A
Bax_R 5′- CAGTTGAAGTTGCCATCAGC - 3′	This study	N/A
Gapdh_F 5′- TGGCAAAGTGGAGATTGTTGCC - 3′	This study	N/A
Gapdh_R 5′- AAGATGGTGATGGGCTTCCCG - 3′	This study	N/A
Trp53inp1_F 5′- AAGTGGTCCCAGAATGGAAGC - 3′	This study	N/A
Trp53inp1_R 5′- GGCGAAAACTCTTGGGTTGT - 3′	This study	N/A
Btg2_F 5′- GTGGGTTTCCTCTCCAGTCTC - 3′	This study	N/A
Btg2_R 5′- CAGTGGTGTTTGTAATGATCGGT - 3′	This study	N/A
Irf4_F 5′- TCCGACAGTGGTTGATCGAC - 3′	This study	N/A
Irf4_R 5′- CCTCACGATTGTAGTCCTGCTT - 3′	This study	N/A
Irf9_F 5′- GCCGAGTGGTGGGTAAGAC - 3′	This study	N/A
Irf9_R 5′- GCAAAGGCGCTGAACAAAGAG- 3′	This study	N/A
Ifit1_F 5′- CTGAGATGTCACTTCACATGGAA - 3′	This study	N/A
Ifit1_R 5′- GTGCATCCCCAATGGGTTCT - 3′	This study	N/A
Ifit3_F 5′- CCTACATAAAGCACCTAGATGGC - 3′	This study	N/A
Ifit3_R 5′- ATGTGATAGTAGATCCAGGCGT - 3′	This study	N/A
Cxcl9_F 5′- GGAGTTCGAGGAACCCTAGTG - 3′	This study	N/A
Cxcl9_R 5′- GGGATTTGTAGTGGATCGTGC - 3′	This study	N/A
Cxcl10_F 5′- CCAAGTGCTGCCGTCATTTTC - 3′	This study	N/A
Cxcl10_R 5′-GGCTCGCAGGGATGATTTCAA - 3′	This study	N/A
Cxcl12_F 5′- TGCATCAGTGACGGTAAACCA - 3′	This study	N/A
Cxcl12_R 5′- TTCTTCAGCCGTGCAACAATC - 3′	This study	N/A
Ccl11_F 5′- GAATCACCAACAACAGATGCAC - 3′	This study	N/A
Ccl11_R 5′- ATCCTGGACCCACTTCTTCTT - 3′	This study	N/A
Ccl25_F 5′- TTACCAGCACAGGATCAAATGG - 3′	This study	N/A
Ccl25_R 5′- CGGAAGTAGAATCTCACAGCAC - 3′	This study	N/A
Recombinant DNA		
pGL3-p21-luc	*Chen*et al.	N/A
MIGR1-human p53, various mutants	Addgene	N/A

(*Continued on next page*)

Continued

REAGENT or RESOURCE	SOURCE	IDENTIFIER
Software and algorithms		
FastQC	Babraham Institute	https://www.bioinformat-ics.babraham.ac.uk/projects/fastqc/
Cutadapt	*Martin*et al.[45]	https://cutadapt.readthedocs.io/en/stable/
HISAT2	*Kim*et al.[46]	http://www.ccb.jhu.edu/software/hisat/
HTSeq	*Anders*et al.[47]	http://www-huber.embl.de/HTSeq
edgeR	*Robinson*et al.[48]	https://bioinf.wehi.edu.au/edgeR/ https://bioconductor.org/packages/edgeR
GSEA	GSEA software	https://www.gsea-msigdb.org/gsea/index.jsp
CellRanger	10x Genomics	https://support.10xgenomics.com/single-cell-gene-expression/software
CellphoneDB	*Lorenzo*et al.	https://github.com/ventolab/CellphoneDB
cBioPortal	*Cerami*et al.[49]	https://www.cbioportal.org/
FlowJo	BD Biosciences	https://www.flowjo.com/
ImageJ	ImageJ software	https://imagej.net/ij/
GraphPad Prism	GraphPad Software	https://www.graphpad.com/
GFOLD	*Feng*et al.[50]	https://zhanglab.tongji.edu.cn/softwares/GFOLD/index.html
Seurat	Seurat	https://satijalab.org/seurat/

EXPERIMENTAL MODEL AND STUDY PARTICIPANT DETAILS

p53-R279W knockin mice and the animals used

The R279W mutant allele of p53 was generated using CRISPR/Cas9 system. Polymerase chain reaction (PCR) was performed using genomic DNA from tail biopsies. The primers used were TCT GTT CCA CGA GTC CCG CC and GGC ATG CGA CTC TCC AGC CTT. PCR products were confirmed by Sanger sequencing for genotyping. $p53^{-/-}$ mice were purchased from GemPharmatech (Nanjing, China). 6–7 weeks adult female C57BL/6 mice (RRID: IMSR_JAX:000664) and $Rag1^{-/-}$ mice with C57BL/6 background (#C001197), weighing 19–23 g, were purchased from Saiye Biotechnology Company. C57BL/6 CD45.1 mice were presented by Dan Liu. The use of mice was approved by the Animal Ethics Committee of the School of Medicine, Shanghai Jiaotong University. All animal care protocols and experiments were reviewed and approved by the Animal Use Committee of the School of Medicine, Shanghai Jiaotong University.

Cell lines and cell culture

U937 cells (ATCC Cat# CRL-1593, RRID: CVCL_0007) were purchased from ATCC. All cell lines were cultured at 37°C in either DMEM or 1640 medium (GIBCO BRL, Invitrogen) supplemented with 10% fetal bovine serum (FBS), 100 U/mL penicillin, and 100 mg/mL streptomycin. All cell lines were tested to be mycoplasma free.

Survival study of W/W mice

For the survival study of W/W mice, p53-R279W knockin mice were monitored twice weekly and euthanized when animals appeared moribund or when the largest palpable tumor reached 2 cm^3 or ulcerated. At day 90 after birth, homozygous ($p53^{R279W/R279W}$, W/W) mice drink water with ATO (35 mg ATO in 1L drinking water) daily until death.

Isolation of primary tumor cells

p53-R279W mice were euthanized with isoflurane and placed on the dissection pad. In the case of lymphoma, the thoracic cavity was opened, and the tumor was separated from the rest of the organs and transferred to cold PBS kept on ice. Gently trisect the tumor with the rubber stopper of a 5 mL syringe and remove large tumor masses. Lymphoma medium (DMEM supplemented with 10% FBS, L-glutamine, penicillin/streptomycin) was added, and cells were passed through a 40 mm nylon cell strainer (Falcon) to remove cell clumps. Cells were spun down and resuspended in a lymphoma medium. Primary sarcoma cells were isolated similarly. Freshly isolated live lymphoma cells or sarcoma cells were counted and either frozen or immediately used for *in vitro* studies or transplantations (allografts).

METHOD DETAILS

Luciferase reporter assay

Luciferase reporter assay was performed as previously reported.[44] Briefly, H1299 cells were transfected with plasmids (p53-R279W-expressing plasmid, luciferase reporter plasmid, Renilla plasmid) using FuGENE transfection reagent (Promega, E1960). After transfection for 24 h, ATO was added, and cells were lysed the next day, followed by luciferase signal determination using a luciferase assay kit (Vazyme, DL101-01).

Immunohistochemistry and immunoblotting

Paraffin-embedded tumor tissue sections were blocked for immunohistochemical analysis using 3% BSA. Subsequently, the sections were stained with primary and secondary antibodies. The primary and secondary antibodies used were rabbit polyclonal antibody Ki-67 (GB111141) and biotinylated goat anti-rabbit immunoglobulin (Servicebio Cat# GB23303, RRID: AB_2811189), respectively. To quantify Ki-67 expression, Ki-67 positive cells were calculated using ImageJ software (RRID: SCR_003070) under ×400 magnification in five randomly selected areas in each tumor sample. TUNEL-positive cells were calculated similarly. For immunoblottings, total protein lysates of tumors or tumor cells were prepared using RIPA buffer (Beyotime, P0013B) containing protease inhibitors. total protein (20 mg) was detected with the following antibodies: Cdkn1a (Santa Cruz Biotechnology Cat# sc-817, RRID: AB_628072), p53 (Abcam Cat# ab26, RRID: AB_303198), β-actin (Santa Cruz Biotechnology Cat# sc-47778 HRP, RRID: AB_2714189), Mdm2 (Sigma-Aldrich Cat# M4308, RRID: AB_260523), ISG15 (Proteintech Cat# 15981-1-AP, RRID: AB_2126302), IFIT1 (Proteintech Cat# 23247-1-AP, RRID: AB_2811269) and Gapdh (ABclonal Cat# AC002, RRID: AB_2736879).

Quantitative PCR

Total RNA was isolated using TRIzol reagent (Invitrogen) and 1μg was reverse-transcribed using HiScript II Q RT SuperMix for qPCR (+ gDNA wiper) (R223-01, Vazyme Biotech). Real-time PCR was performed in duplicate using ChamQ SYBR qPCR Master Mix (Low ROX Premixed; Q331-02/03, Vazyme Biotech) and a ViiATM 7 Real-time PCR System (Applied Biosystems) under the following conditions: 10 min at 95°C, followed by 40 cycles of 95°C for 15 s and 60°C for 60 s. The following primer sets for mouse sequences were used: *Cdkn1a*_F 5′- CCTGGTGATGTCCGACCTG - 3′, *Cdkn1a*_R 5′- CCATGAGCGCATCGCAATC - 3′, *Mdm2*_F 5′- GCGT GGAATTTGAAGTTGAGTC - 3′, *Mdm2*_R 5′- CTGTATCGCTTTCTCCTGTCTG - 3′, *Bbc3*_F 5′- CCTCTGGAAGTGCTGGGAAG - 3′, *Bbc3*_R 5′- CTCCCTGGAGCCTATGGGT - 3′, *Bax*_F 5′- TTGGAGATGAACTGGACAGC - 3′, *Bax*_R 5′- CAGTTGAAGTTGCCA TCAGC - 3′, *Gapdh*_F 5′- TGGCAAAGTGGAGATTGTTGCC - 3′, *Gapdh*_R 5′- AAGATGGTGATGGGCTTCCCG - 3′, *Trp53inp1*_F 5′- AAGTGGTCCCAGAATGGAAGC - 3′, *Trp53inp1*_R 5′- GGCGAAAACTCTTGGGTTGT - 3′, *Btg2*_F 5′- GTGGGTTTCCTCTCCAG TCTC - 3′, *Btg2*_R 5′- CAGTGGTGTTTGTAATGATCGGT - 3′, *Irf4*_F 5′- TCCGACAGTGGTTGATCGAC - 3′, *Irf4*_R 5′- CCTCAC GATTGTAGTCCTGCTT - 3′, *Irf9*_F 5′- GCCGAGTGGTGGGTAAGAC - 3′, *Irf9*_R 5′- GCAAAGGCGCTGAACAAAGAG- 3′, *Ifit1*_F 5′- CTGAGATGTCACTTCACATGGAA - 3′, *Ifit1*_R 5′- GTGCATCCCCAATGGGTTCT - 3′, *Ifit3*_F 5′- CCTACATAAAGCACCTA GATGGC - 3′, *Ifit3*_R 5′- ATGTGATAGTAGATCCAGGCGT - 3′, *Cxcl9*_F 5′- GGAGTTCGAGGAACCCTAGTG - 3′, *Cxcl9*_R 5′- GGGATTTGTAGTGGATCGTGC - 3′, *Cxcl10*_F 5′- CCAAGTGCTGCCGTCATTTTC - 3′, *Cxcl10*_R 5′- GGCTCGCAGGGAT GATTTCAA - 3′, *Cxcl12*_F 5′- TGCATCAGTGACGGTAAACCA - 3′, *Cxcl12*_R 5′- TTCTTCAGCCGTGCAACAATC - 3′, *Ccl11*_F 5′- GAATCACCAACAACAGATGCAC - 3′, *Ccl11*_R 5′- ATCCTGGACCCACTTCTTCTT - 3′, *Ccl25*_F 5′- TTACCAGCACAGGATCAAA TGG - 3′, *Ccl25*_R 5′- CGGAAGTAGAATCTCACAGCAC - 3'.

High-resolution ultrasound imaging and analysis

Image acquisition was performed with the Vevo LAZR-X US-PAI system (Fujifilm Visual Sonics) and a 75 μm axial resolution probe (MX250S). Mice were sedated with 5% isoflurane/oxygen (vol/vol), and anesthesia was maintained with 1–2% (vol/vol) isofluorane. For lymphoma recording, we optimized the sweep speed, depth, focus, and gain settings to obtain the best possible images. The anesthetized mice were placed on a heated imaging table maintained at a temperature of approximately 37°C. Images of parallel and vertical mice thymus were obtained. After image acquisition, the mice were returned to clean cages for safe recovery post-imaging. Tumor dimensions [length (L), width (W), and height (H)] with digital calipers and photographs were obtained. Tumor volume was calculated using the formula: $(L \times W \times H \times \pi)/6$. The maximum regression ratio was calculated using the formula: (maximum regression volume − Initial volume)/Initial volume. Starting from day 70 after birth, high-resolution ultrasound imaging was conducted 1–2 times every week. On day 90, mice with T-lymphoma tumor volumes ranging from 50 to 100 mm^3 were selected and randomly grouped. During ATO treatment, ultrasound imaging was performed once every 3–4 days until the mice died. After their demise, the intervals for ultrasound imaging were extended.

Allograft model study

For allograft transplantation, 5×10^6 primary lymphoma cells suspended in 100 μL saline were injected subcutaneously (SC) into C57BL/6N or $Rag1^{-/-}$ mice on day 0. On day 7, the tumors had grown to a certain size, and the tumor-bearing mice were randomly grouped into control and treatment groups. In the control group, mice were intraperitoneally injected with PBS daily. In the ATO treatment group, mice were intraperitoneally injected with 5 mg/kg ATO daily. For $CD4^+$, $CD8^+$ T cell, and IFN-γ depletion, 200 μg of antibodies were injected intraperitoneally every three days starting from day 7. The control group received the same amount of isotype

control antibody. Tumor volumes were measured at least twice weekly and calculated as 0.5 × lengths × width × height. The tumor growth inhibition (TGI) value was calculated using the formula: ($Volume_{PBS}$ – $Volume_{ATO}$)/$Volume_{PBS}$ and ($Weight_{PBS}$ – $Weight_{ATO}$)/ $Weight_{PBS}$.

RNA-sequencing

Raw reads were initially processed using FastQC (v0.10.1, RRID: SCR_014583) for quality control, and adapter sequences and poor-quality reads were removed using Cutadapt (v1.9.1, RRID: SCR_011841). Quality-filtered reads were aligned to the Mus musculus genome (GRCm38) using HISAT2 (v2.0.1, RRID: SCR_015530). Principal Component Analysis (PCA) was performed in R Studio using the prcomp function. Gene-level read counts were generated from aligned reads using HTSeq (v0.6.1, RRID: SCR_005514), and differential gene expression analysis was performed using the R (v4.2.2) edgeR package (v 3.40.2, RRID: SCR_012802). Genes with a fold change ≥ 2 or ≤ 0.5 and $p < 0.05$ were considered differentially expressed. Volcano plots and heatmaps were generated using R with ggplot2 (v.3.4.1, RRID: SCR_014601) and pheatmap (v1.0.12, RRID: SCR_016418), respectively. To understand the function of the DEGs, pathway enrichment analysis was performed using the g: profiler website (https://biit.cs.ut.ee/gprofiler/gost/gost). GSEA software (v4.1.0, RRID: SCR_003199) was used to determine whether a priori-defined gene set showed a statistically significant difference between different groups of samples. GSEA was performed using the gene set collection hallmark (50 gene sets) downloaded from the Molecular Signature Database (MSigDB; v7.4; http://software. broadinstitute.org/gsea/msigdb).

Flow cytometric analysis

Tumors were cut into small pieces and mechanically disassociated into single-cell suspensions in PBS supplemented with 2% bovine serum albumin (BSA). And then the cell suspension was incubated with antibodies for 30 min at 4°C in the dark. The cells were washed and filtered through a 70-micron filter before flow cytometric analysis using a BD LSRFortessa. An example of gating schematics is shown in Figure S4D to characterize immune cell infiltrates. Antibodies: CD45.1-FITC (BioLegend Cat# 110705, RRID: AB_313494), CD3-PE/Cyanine7 (BioLegend Cat# 100219, RRID: AB_1732068), CD19-Brilliant Violet 510 (BioLegend Cat# 115545, RRID: AB_2562136), CD4-FITC (BioLegend Cat# 100405, RRID: AB_312690), CD4-APC/Cyanine7 (BioLegend Cat# 100413, RRID: AB_312698), CD8a-PE (BioLegend Cat# 100707, RRID: AB_312746), CD11B-PE/Cyanine7 (BioLegend Cat# 101215, RRID: AB_312798), CD11C-PerCP (BioLegend Cat# 117325, RRID: AB_893236, F4/80-Brilliant Violet 510 (BioLegend Cat# 123135, RRID: AB_2562622), NK1.1-APC (BioLegend Cat# 156505, RRID: AB_2876525).

ScRNA-seq of patient's PBMCs

The scRNA-seq data were compiled from the GSE182926 dataset (https://doi.org/10.1126/scitranslmed.abn9155). In brief, PBMCs harvested at diagnosis and after ATO treatment were obtained from the sample repository of an ongoing clinical trial. The raw sequencing reads of samples were aligned to the human ENSEMBL GRCh38 reference using CellRanger (10× Genomics, default settings, version 6.0.0, RRID: SCR_021002). Unsupervised clustering and cell annotation were performed as previously reported.[21] The DEGs between at-diagnosis PBMCs and treated PBMCs were identified using filtration criteria as FC $\geq$ 1.5 or $\leq$0.67 and $p \geq 10^{-20}$ and then enrolled for the enrichment analysis using the g: profiler website (https://biit.cs.ut.ee/gprofiler/gost/gost). Cell-cell interactions were analyzed using Systematic CellphoneDB (RRID: SCR_017054).[35]

Proteomics profiling

Tumor tissues were homogenized in RIPA buffer containing protease inhibitors and 100 mM PMSF. Protein lysates were processed using the filter-assisted sample preparation (FASP) protocol.[51] The processed samples were dried and resuspended for LC-MS/MS analysis. The raw data were analyzed using the Mus musculus protein database (UniProt, 2022.01.11) and processed with DIA-NN v1.8 (https://github.com/vdemichev/DiaNN/releases/tag/1.8.1) with default parameters.[52] Up to two missed cleavages were allowed. A 1% false discovery rate (FDR) threshold was applied for both protein and peptide identifications.

TCGA Pan-Cancer Atlas data analysis

The RNA-seq gene expressions, clinical characteristics, and mutation information of TCGA Pan-Cancer Atlas were downloaded from UCSC Xena (https://xenabrowser.net/) using the R package UCSCXenaTools.[53] TCGA PanCanAtlas data including 9,875 primary tumor samples from 33 tumor types were evaluated.[54,55] To quantify the gene expression levels, the Fragments Per Kilobase of transcript per Million mapped reads (FPKM) metric was employed. For the identification of p53 mutations, mutation calling results from four different pipelines were utilized: MuSE,[56] MuTect,[57] SomaticSniper,[58] and VarScan,[59] which helps to ensure the accuracy of p53 mutation detection. The immune scores of TCGA PanCanAtlas were calculated using the R package ESTIMATE.[60] Additionally, to estimate T cell abundance within the tumors, single sample gene set enrichment analysis (ssGSEA) [61] was performed. T cell markers from tumor-infiltrating T cells obtained across the Pan-Cancer cohorts were used to provide a detailed assessment of T cell presence in the tumor microenvironment.[36]

QUANTIFICATION AND STATISTICAL ANALYSIS

Statistics analysis (unpaired two-tailed Student's t test, with a 95% confidence interval under the untested assumption of normality) was performed using Prism 9 (GraphPad, RRID: SCR_002798). Error bars in the Figures indicate the SD of the number of replicates. Kaplan–Meier analysis was used to calculate the survival rate, and the log rank test was used to determine the significance of differences between survival curves. Hazard ratios (HR) and 95% confidence intervals (CI) were used to assess the risk ratio of death. The significant differences between the two groups were noted by asterisks (*$p < 0.05$, **$p < 0.01$, ***$p < 0.001$, ****$p < 0.0001$).

Cell Reports Medicine, Volume *6*

Supplemental information

Pharmacological rescue of mutant p53 triggers spontaneous tumor regression via immune responses

Jiabing Li, Shuang Zhang, Baohui Wang, Yuting Dai, Jiale Wu, Dianjia Liu, Ying Liang, Shujun Xiao, Zhengyuan Wang, Jiaqi Wu, Derun Zheng, Xueqin Chen, Fangfang Shi, Kai Tan, Xianting Ding, Huaxin Song, Sujiang Zhang, and Min Lu

SUPPLEMENTARY INFORMATION

Figure S1

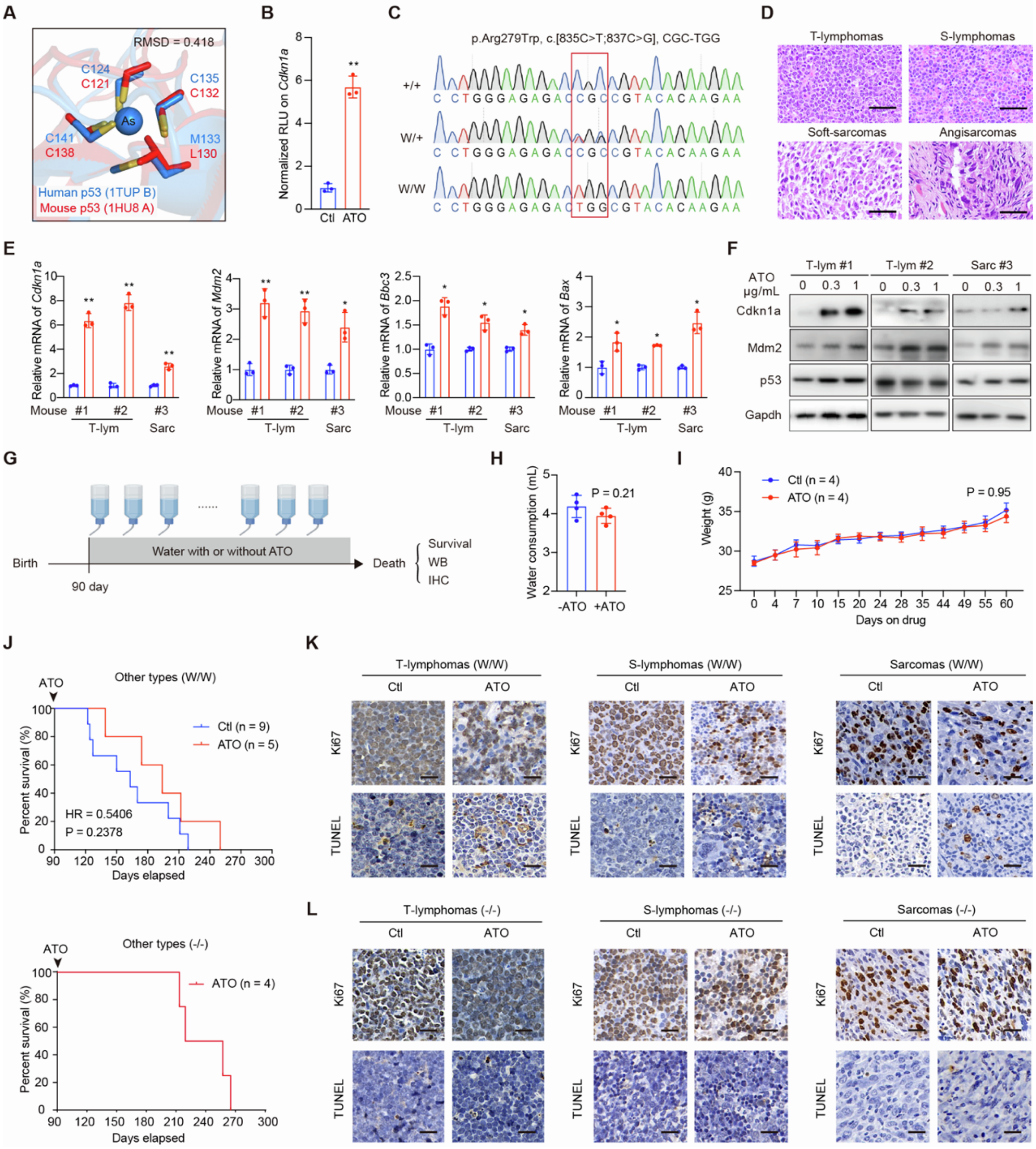

Figure S1. Pharmacologically rescued p53-R279W extended mice survival. Related to Figure 1.

(A) Close-up view of ABP-equivalent residues in the human wild-type p53 (1TUP chain B) and mouse wild-type p53 (1HU8 chain A) crystal structures. The ABP-equivalent residues are represented as sticks. The arsenic atom from the arsenic-bound G245S DBD (7DHY chain A) was superimposed onto human p53 and portrayed as a sphere.

The root-mean-square deviation (RMSD) calculation quantifies the differences between ABP residues in humans and mice.

(B) Luciferase reporter assay of mouse p53-R279W transcriptional activity on the mouse *Cdkn1a* promoter in H1299 cells upon treatment with 1 μg/ml ATO for 24h. Bar graphs show normalized relative light units (RLU).

(C) Sanger sequencing peak plots of the indicated genotypes in mice. Mutant codon positions were framed.

(D) H&E staining of the indicated tumors isolated from naturally dead W/W mice. Scale bar, 50 μm.

(E) mRNA levels of *Cdkn1a, Mdm2, Bbc3,* and *Bax* upon 1 μg/ml ATO treatment for 24h in the primary tumor cells derived from three naturally dead W/W mice.

(F) Immunoblotting of the indicated proteins in primary tumor cells derived from W/W mice upon treatment with 0, 0.3, and 1 μg/ml ATO for 48h.

(G) Schematic diagram of ATO treatment as in Figure 1D-1I.

(H) Water consumption by mice in the indicated groups (n = 4 mice per group).

(I) Body weight curves. The body weights of mice were measured every three days (n = 4 mice per group).

(J) Kaplan–Meier survival curves of W/W and −/− mice, with other tumor types, as shown in Figure 1D-1K.

(K-L) Representative immunohistochemical staining images of Ki67 and TUNEL in paraffin-embedded sections of T-lymphomas and S-lymphomas. Scale bars, 50 μm.

Bars represent mean ± SD, unpaired two-tailed Student’s t-test; In I, two-way ANOVA for multiple comparison testing. *P < 0.05, **P < 0.01.

Fig.S2

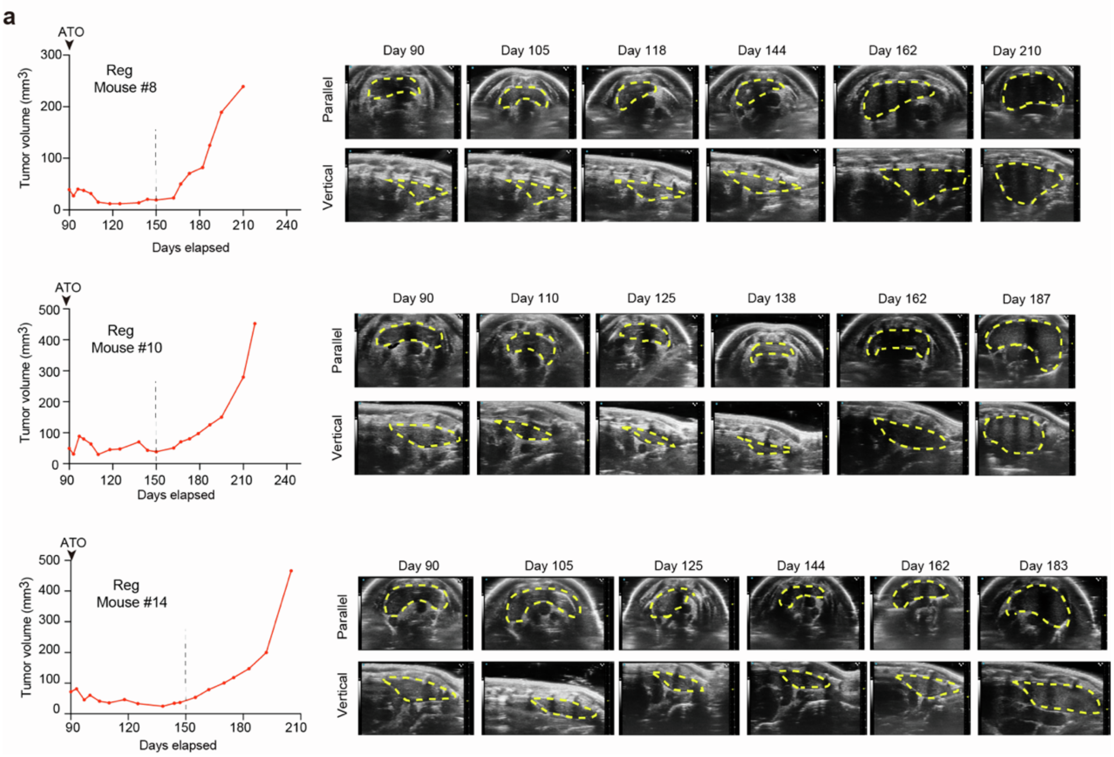

Figure S2. Rescued p53-R279W triggered regression of spontaneous lymphoma. Related to Figure 2.

(A) Examples of parallel and vertical ultrasound images of T-lymphomas in three representative mice from the regression group. The left panel of each image shows the calculated tumor volumes.

Fig.S3

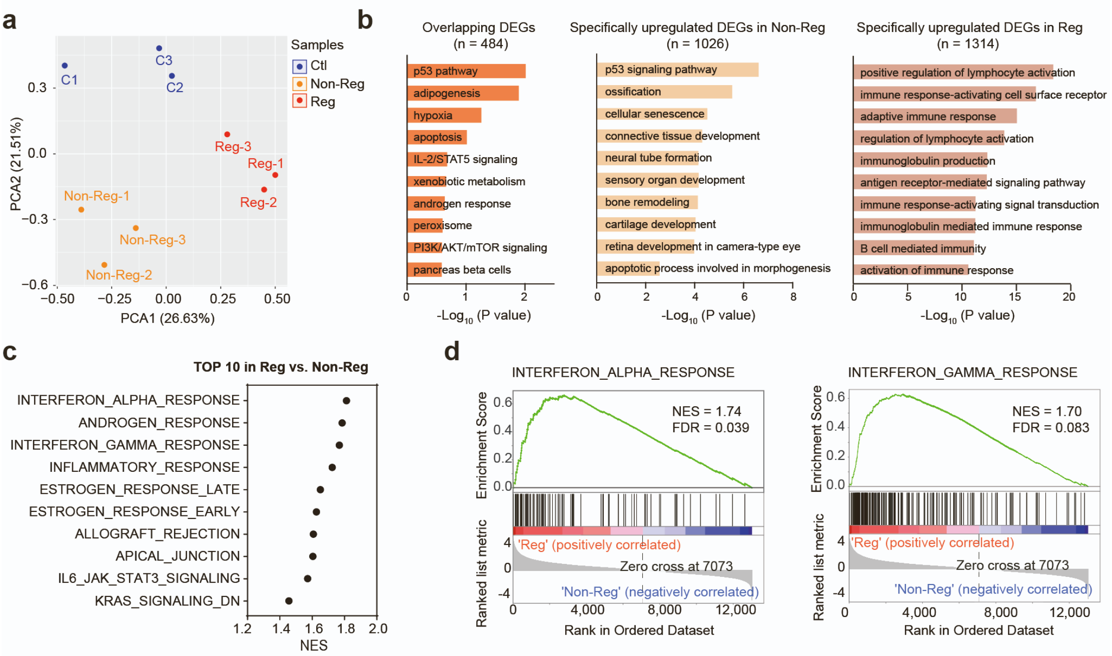

Figure S3. Lymphoma regression is associated with immune response. Related to Figure 3.

(A) 2D score plot of Principal Component Analysis (PCA) of tumors from three groups, as shown in Figure 3A.

(B) The full list of the top ten enriched pathways, as illustrated in Figure 3C.

(C) Dot plot showing the top ten pathways enriched in the Reg group identified by GSEA of hallmark gene sets compared to the Non-Reg group. NES: normalized enrichment score.

(D) GSEA plots of the enriched terms of “interferon alpha response” and “interferon gamma response” in C. NES and FDR (False discovery rate) are shown.

Fig.S4

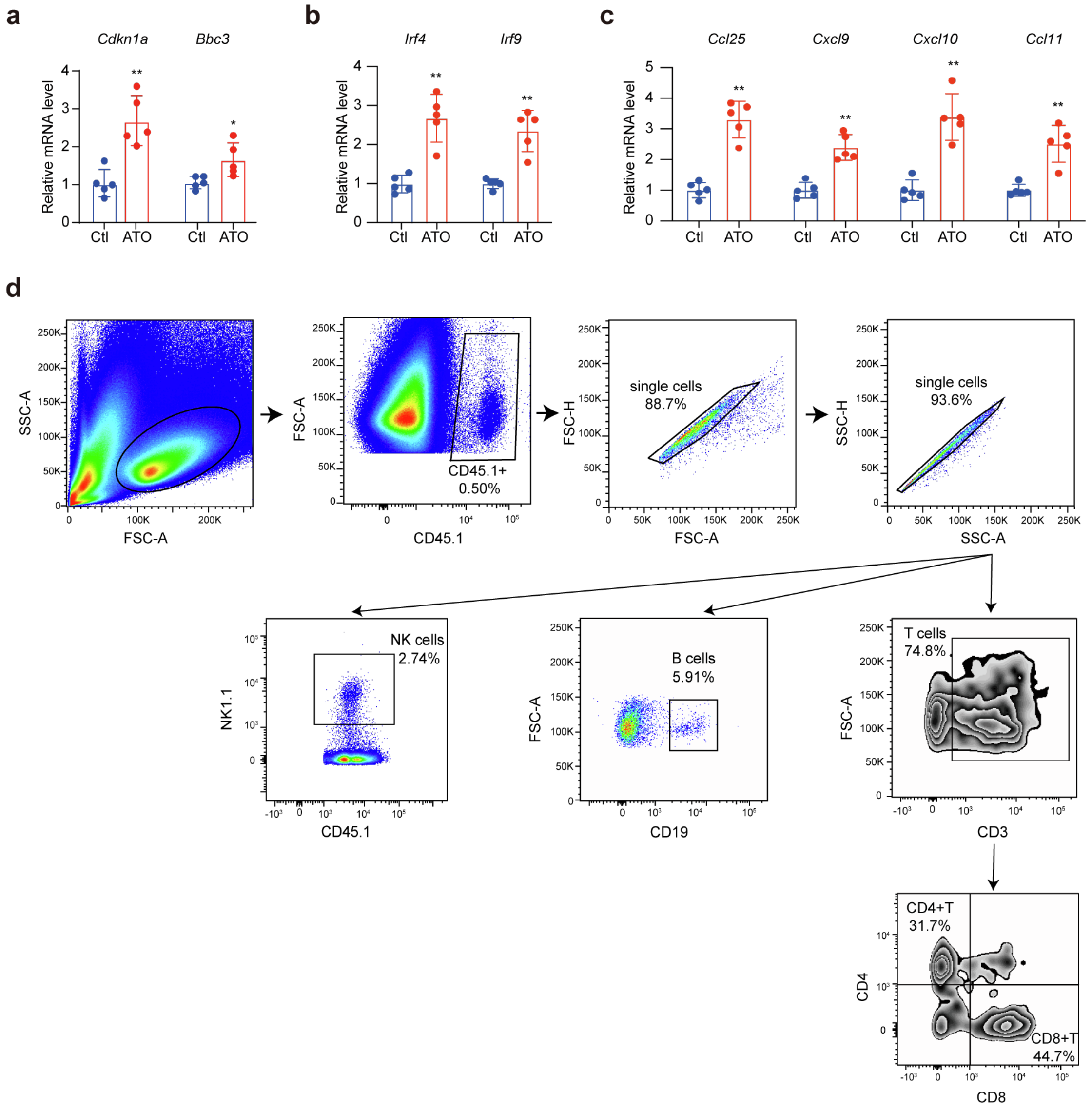

Figure S4. The anticancer immune response is largely dependent on the activation of CD8⁺ T cells. Related to Figure 4.

(A-C) RT-qPCR validation of the relative mRNA levels of the indicated genes in the isolated tumors from Figure 4C. (A) p53 targets. (B) IFN pathway genes. (C) chemokine genes.

(D) Gating strategy of immune cells as in Figure 4E-4G.

Bars represent mean ± SD, unpaired two-tailed Student's t-test, *P < 0.05, **P < 0.01.

Fig.S5

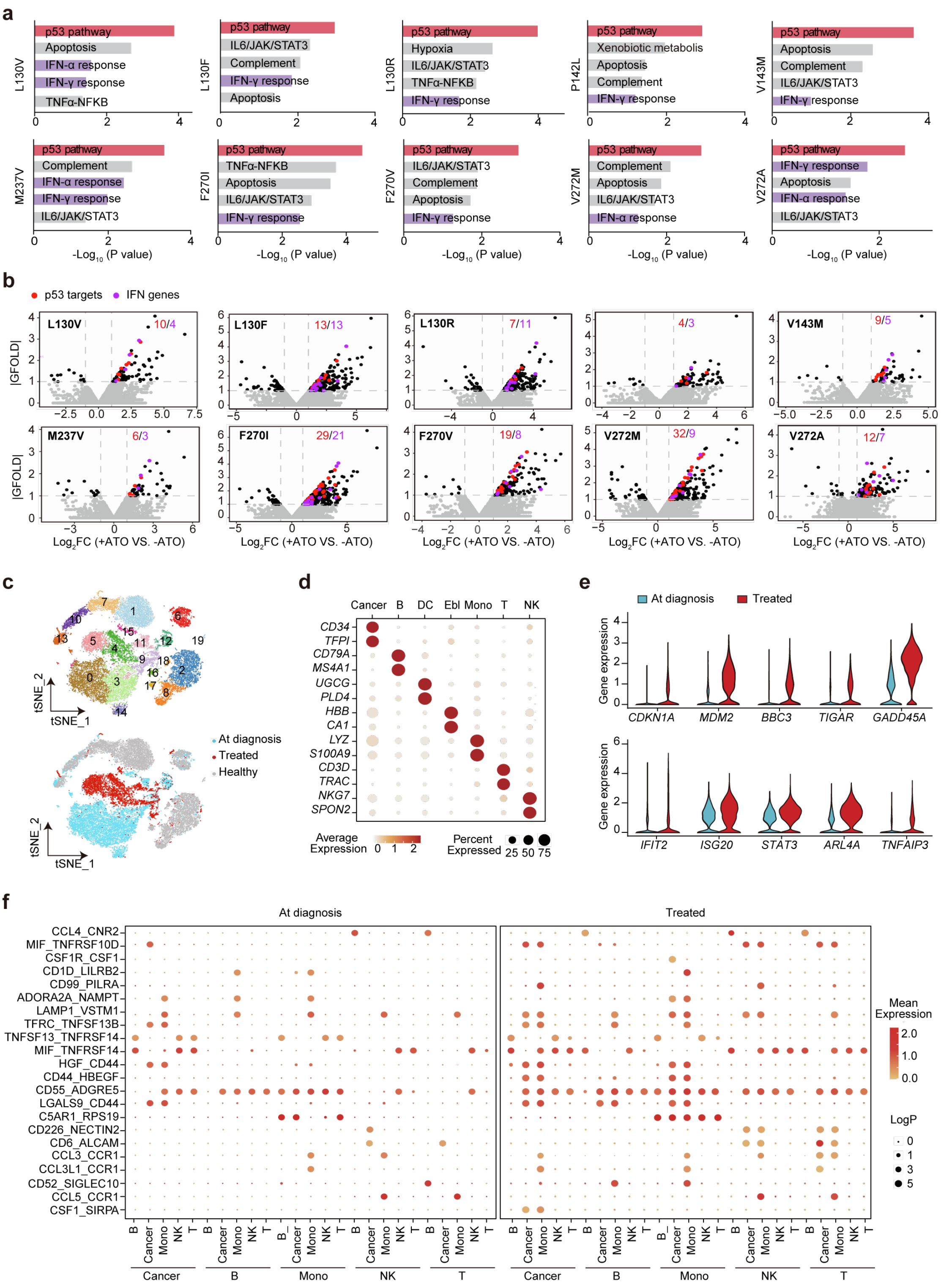

Figure S5. The broad applicability of rescued p53 mutants in triggering immune responses in human cells. Related to Figure 5.

(A) Top five pathways enriched in ATO-treated cells harboring the indicated type-1

p53 mutants. These enriched pathways were identified by the GSEA of hallmark gene sets for each mutant.

(B) Volcano plot showing DEGs (FC ≥ 2 or ≤ 0.5, GFOLD ≥ 1 or ≤ -1, FPKM ≥ 1) of the 10 indicated cell lines upon ATO treatment.

(C) Unsupervised *t*-SNE plot generated from datasets of PBMC samples in at-diagnosis, treated, and healthy humans.

(D) Dot plot of the marker genes for each classified cell type. These markers annotated 19 clusters into the six indicated cell types (cancer, cancer cells; B, B cells; T, T cells; NK, NK cells; Mono, monocytes; Ebl, erythroblasts; DC, dendritic cells).

(E) Violin plots showing the mRNA levels of representative p53 target genes (upper panel) and IFN pathway genes (lower panel) in cancer cells.

(F) Dot plot showing the expression of significant ligand-receptor pairs involved in the interaction among the six cell types before and after ATO treatment. P values are indicated by circle size (permutation test). The means of the average expression levels of interactions are indicated in color. The cell types below the line are ligand cells, and the cell types above the line are the corresponding receptor cells.
